# The genome and lifestage-specific transcriptomes of a plant-parasitic nematode and its host reveal susceptibility genes involved in trans-kingdom synthesis of vitamin B5

**DOI:** 10.1038/s41467-022-33769-w

**Published:** 2022-10-19

**Authors:** Shahid Siddique, Zoran S. Radakovic, Clarissa Hiltl, Clement Pellegrin, Thomas J. Baum, Helen Beasley, Andrew F. Bent, Oliver Chitambo, Divykriti Chopra, Etienne G. J. Danchin, Eric Grenier, Samer S. Habash, M. Shamim Hasan, Johannes Helder, Tarek Hewezi, Julia Holbein, Martijn Holterman, Sławomir Janakowski, Georgios D. Koutsovoulos, Olaf P. Kranse, Jose L. Lozano-Torres, Tom R. Maier, Rick E. Masonbrink, Badou Mendy, Esther Riemer, Mirosław Sobczak, Unnati Sonawala, Mark G. Sterken, Peter Thorpe, Joris J. M. van Steenbrugge, Nageena Zahid, Florian Grundler, Sebastian Eves-van den Akker

**Affiliations:** 1 Department of Entomology and Nematology, University of California Davis, One Shields Avenue, Davis, CA 95616 USA; 2 Rheinische Friedrich-Wilhelms-University of Bonn, INRES - Molecular Phytomedicine, Karlrobert- Kreiten-Straße 13, D-53115 Bonn, Germany; 3 The Crop Science Centre, Department of Plant Sciences, University of Cambridge, Cambridge, CB2 3EA UK; 4 Department of Plant Pathology and Microbiology, Iowa State University, Ames, IA 50011 USA; 5 Department of Plant Pathology, University of Wisconsin—Madison, Madison, WI 53706 USA; 6 Université Côte d’Azur, INRAE, CNRS, Institut Sophia Agrobiotech, Sophia-Antipolis, France; 7 IGEPP, INRAE, Institut Agro, Université Rennes, 35650 Le Rheu, France; 8 Laboratory of Nematology, Wageningen University & Research, Droevendaalsesteeg 1, 6708PB Wageningen, The Netherlands; 9 Department of Plant Sciences, University of Tennessee, Knoxville, TN 37996 USA; 10 Department of Botany, Institute of Biology, Warsaw University of Life Sciences (SGGW), Nowoursynowska 159, 02-787 Warsaw, Poland; 11 Genome Informatics Facility, Iowa State University, Ames, IA 50010 USA; 12 Mackenzie Institute for Early Diagnosis, School of Medicine, University of St Andrews, North Haugh, St Andrews, KY16 9TF UK; 13 Institute for Microbiology and Biotechnology, Rheinische Friedrich-Wilhelms-University of Bonn, Meckenheimer Allee 168, D-53115 Bonn, Germany; 14 Present Address: BASF Vegetable Seeds, Napoleonsweg 152, 6083 AB Nunhem, The Netherlands; 15 Present Address: P.H. Petersen Saatzucht Lundsgaard GmbH, D-24977 Grundhof, Germany; 16 Present Address: Solynta, Dreijenlaan 2, 6703 HA Wageningen, The Netherlands

**Keywords:** Parasitism, Transcriptomics, Genomics, Agroecology

## Abstract

Plant-parasitic nematodes are a major threat to crop production in all agricultural systems. The scarcity of classical resistance genes highlights a pressing need to find new ways to develop nematode-resistant germplasm. Here, we sequence and assemble a high-quality phased genome of the model cyst nematode *Heterodera schachtii* to provide a platform for the first system-wide dual analysis of host and parasite gene expression over time, covering all major parasitism stages. Analysis of the hologenome of the plant-nematode infection site identified metabolic pathways that were incomplete in the parasite but complemented by the host. Using a combination of bioinformatic, genetic, and biochemical approaches, we show that a highly atypical completion of vitamin B5 biosynthesis by the parasitic animal, putatively enabled by a horizontal gene transfer from a bacterium, is required for full pathogenicity. Knockout of either plant-encoded or now nematode-encoded steps in the pathway significantly reduces parasitic success. Our experiments establish a reference for cyst nematodes, further our understanding of the evolution of plant-parasitism by nematodes, and show that congruent differential expression of metabolic pathways in the infection hologenome represents a new way to find nematode susceptibility genes. The approach identifies genome-editing-amenable targets for future development of nematode-resistant crops.

## Abstract (web summary)

Plant-parasitic nematodes are a threat to crop production. Combining bioinformatics, genetic and biochemical approaches, the authors show that the plant pathogen beet cyst nematode possesses an incomplete vitamin B5 synthesis pathway, of potential prokaryotic origin, complemented by its plant host.

## Introduction

Plant-parasitic nematodes are a major threat to crop production systems all around the world, and in some crops (e.g., soybean) are the dominant pathogen of any kind^1, 2^. They are estimated to cause losses of up to 25% each year, with a value of >80 billion US dollars^3^. The major contributors to these losses are notoriously difficult-to-control obligate biotrophic sedentary endo-parasites: a remarkable group of parasites that live inside, and feed on living host root tissue.

Cyst nematodes, one of the two major groups of sedentary endo-parasites, are a perennial constraint on crop production. Their control is difficult due to their complex biology. A cyst, formed from the body wall of a dead female, contains hundreds of eggs that can remain dormant in the soil for many years. Eggs contain a fully-formed, but sexually undetermined, second-stage juvenile (J2) that will hatch in response to various host-derived cues^4^. Hatching is a critical decision: the J2 will migrate through the soil to find a host, invade the root through a combination of a mechanical disruption using a needle-like stylet and enzymatic secretions, and move towards the vascular cylinder—all without feeding. Once J2s reach the vascular cylinder, their behaviour changes (stylet thrusts become more exploratory), and a single cell is chosen for feeding site development^5^. To manipulate plant development and immunity, the nematode injects a cocktail of proteins and other molecules, termed effectors into the plant cell cytoplasm^6^ and surrounding apoplast^7^. A majority of these effectors originate from two sets of gland cells (one dorsal and two subventral) and cause an existing plant cell, often procambial, to re-differentiate into a syncytial feeding site that is unlike any other tissue in the plant^8^.

Host cell manipulation is rapid and profound: within just a few days, the cell cycle arrests at G2 phase, the vacuole reduces in size and fragments, the nucleus greatly enlarges, the cytoplasm becomes enriched in organelles by an extensive proliferation of the rough and smooth endoplasmic reticulum, ribosomes, mitochondria and plastids (chloroplasts and amyloplasts). The cell wall is locally degraded to promote protoplast fusion with hundreds of adjacent cells in an iterative manner^9–11^. The establishment of the feeding site is a point of no return: concurrent with establishing the feeding site, cyst nematodes become sedentary. If at any time during the interaction the syncytium dies, so does the nematode^12^.

Unusually for an animal, cyst nematode sex is determined during parasitism. It is understood that the sexually undetermined juvenile with access to ample nutrition (from a well-formed feeding site) will preferentially develop into a female, while those with limited nutrition preferentially develop into males, stop feeding, and regain motility and search for a mate^13, 14^. The obligate sexual cyst nematode females will ultimately fill with eggs, their body wall will tan and harden, and the resultant cyst will drop off the root, and their eggs can remain dormant in the soil for several years.

Cyst nematodes (particularly the *Heterodera* genus) are notoriously difficult-to-control due, at least in part, to the relative scarcity of commercially viable resistance sources. Resistance can be achieved by either gain-of-function resistance genes (plant genes that block the parasite/feeding site development in some way), or loss-of-function of susceptibility genes (plant genes that are necessary for feeding site development/nematode infection). Given the relative scarcity of classical resistance genes against Heterodera^15, 16^ there is a pressing need to better understand the biology of host infection and identify nematode susceptibility genes. The beet cyst nematode, *Heterodera schachtii*, is the obvious choice in which to build this understanding because: (a) it can infect the model plant *Arabidopsis thaliana*, (b) it is an important agricultural pest on sugar beet and brassicaceous crops in its own right, and (c) its close relative the soybean cyst nematode, *Heterodera* *glycines*, is one of the severely economically damaging plant-parasitic nematodes worldwide.

Here we sequence and assemble a high-quality genome of the beet cyst nematode *H. schachtii* to provide a platform for the first system-wide and simultaneous analysis of host and parasite gene expression across time, covering all major stages of the interaction. To focus on novel aspects of biotrophy that may reveal nematode susceptibility genes, we analysed metabolic pathways that were incomplete in the parasite, but complemented by the host. Using a combination of bioinformatic, genetic, and biochemical approaches, we highlight the highly atypical completion of the vitamin B5 (pantothenate) biosynthesis pathway by the nematode parasite that was most likely enabled by a horizontal gene transfer from a bacterium. Importantly, knockout of either the plant-encoded or the now nematode-encoded steps in the pathway blocks parasitism. Our experiments establish a reference for cyst nematodes, use this as a platform to further our fundamental understanding of the evolution of plant-parasitism, and show that the understanding of the congruent differential expression of metabolic pathways represents a new way to find nematode susceptibility genes, and thereby targets for the generation of genome edited crops resistant to nematodes.

## Results and discussion

### Sequencing and assembly of the *H. schachtii* genome

We measured (Supplemental Fig. 1), sequenced (BioProject PRJNA722882), and assembled the genome of *H. schachtii* (population Bonn) using a combination of flow cytometry, Pacific Biosciences sequencing, and Illumina sequencing. *H. schachtii* has the largest genome (160–170 Mb) of any cyst nematode measured/sequenced to date (Supplementary Table 1). It was sequenced to 192-fold coverage using Pacific Biosciences sequencing (fragment n50 of 16 kb), and 144-fold coverage using Illumina sequencing (150 bp Paired-end reads). The final, polished, contamination-free (Supplemental Fig. 2), assembly (v1.2) included ~179 Mbp contained within 395 scaffolds: 90% of the sequence is contained on scaffolds longer than 281,463 bp (*n* = 154). The assembly is a largely complete haploid representation of the diploid genome, as evidenced by core eukaryotic genes being largely present, complete and single copy (CEGMA 93.15% complete with an average of 1.12 copies each, and BUSCO (Eukaryota odb9) 79% complete with 8.2% duplicated—Supplementary Table 2). Over three million variants were phased into haplotypes (2029 blocks, N50 239.5 kb, covering 94.7% of the reference) which can be used to predict true protein variants (Supplementary data 1), and 601 larger structural variants were identified (Supplementary data 2).

### The trans-kingdom, lifestage-specific, transcriptomes of *H. schachtii* and *A. thaliana* provide a holistic view of parasitism

**Fig. 1:**
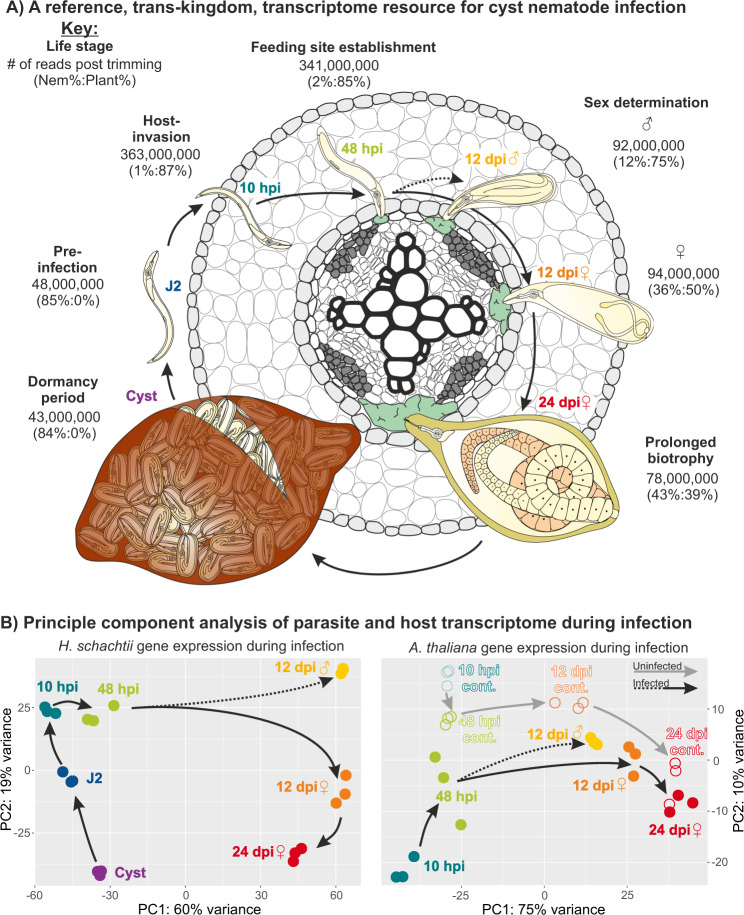
Trans-kingdom, lifestage-specific, transcriptome of *H. schachtii* and *A. thaliana*. **A** Schematic representation of the life cycle of *H. schachtii* infecting *A. thaliana*, highlighting the 7 stages sampled in this study. For each stage, the average number of trimmed RNAseq read pairs per replicate is shown, with the proportion of reads mapping to either parasite or host in parentheses. **B** Principle components 1 and 2 for *H. schachtii* and *A. thaliana* expression data are plotted. Arrows indicate progression through the life cycle/real-time. Hours post infection (hpi), days post infection (dpi).

We devised a sampling procedure to cover all major life stages/transitions of the parasitic life cycle to generate a simultaneous, chronological, and comprehensive picture of nematode gene expression, and infection-site-specific plant gene expression patterns. We sampled cysts and pre-infective second-stage juveniles (J2s), as well as infected segments of *A. thaliana* root and uninfected adjacent control segments of root at 10 hours post infection (hpi - migratory J2s, pre-establishment of the feeding site), 48 hpi (post establishment of the feeding site), 12 days post infection females (dpi - virgin), 12 dpi males (differentiated, pre-emergence, most if not all stopped feeding), and 24 dpi females (post mating), each in biological triplicate (Fig. 1A). We generated approximately nine billion pairs of 150 bp strand-specific RNAseq reads (Supplementary data 3) covering each stage in biological triplicate (for the parasite and the host): in the early stages of infection we generated over 400 million reads per replicate, to provide sufficient coverage of each kingdom.

Strand-specific RNAseq reads originating from host and parasite were deconvoluted by mapping to their respective genome assemblies (*H. schachtii* v.1.2 and TAIR10). For the parasite, ~500 million Illumina RNAseq read pairs uniquely mapping to the *H. schachtii* genome were used to generate a set of 26,739 gene annotations (32,624 transcripts - detailed further in the next section), ~77% of which have good evidence of transcription in at least one lifestage (≥10 reads in at least one rep). Similarly for the host, ~2.8 billion Illumina RNAseq read pairs uniquely mapping to the *A. thaliana* genome show that ~77% of the 32,548 gene models have good evidence of transcription in at least one stage (≥10 reads in at least one rep, even though we only sampled roots). A principal component analysis of the host and parasite gene expression data offers several insights into the parasitic process. Principle component 1 (60% of the variance) and 2 (19% of the variance) of the parasite recapitulate the life cycle in PCA space (Fig. 1B). The 12 dpi female transcriptome is more similar to the 24 dpi female transcriptome than to the 12 dpi male transcriptome. Principle components 1 (75% of the variance) and 2 (10% of the variance) of the host show that the greatest difference between infected and uninfected plant tissue is at the early time points (10 hpi), and that the transcriptomes of infected and uninfected plant material converge over time, possibly due to systemic effects of infection. A 12 dpi male syncytium transcriptome is roughly intermediate between a control root transcriptome and a 12 dpi female syncytium transcriptome. Given that at this stage most if not all of the males will have ceased feeding, this could be due to inadequate formation of the feeding site, or regression of the tissue. In any case, by comparing both principal component analyses, we can see that what is a relatively small difference in the transcriptomes of the feeding sites of males and females is amplified to a relatively large difference in the transcriptomes of the males and females themselves (Fig. 1B).

### The consequences, and possible causes, of large-scale segmental duplication in the Heterodera lineage

**Fig. 2:**
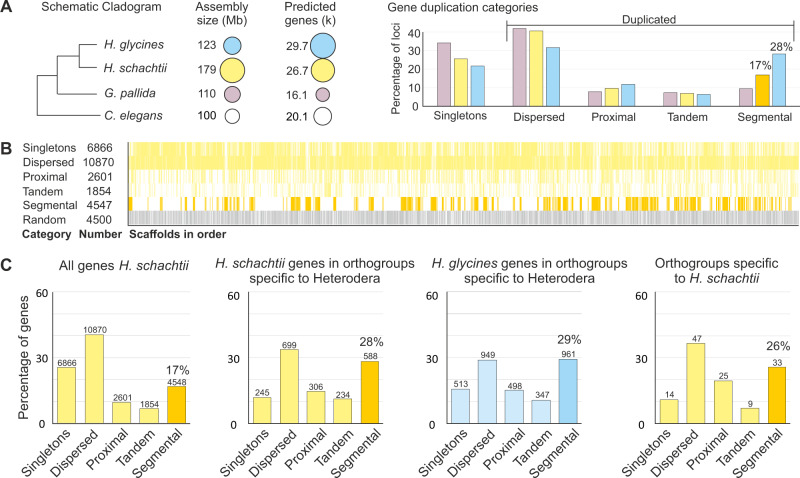
Large-scale segmental duplications in the Heterodera lineage acted on old and new genetic capital. **A** Schematic cladogram, assembly size, number of predicted genes, and gene duplication categories are shown for *H. schachtii* (yellow), *H. glycines* (blue), *G. pallida* (purple), and *C. elegans* (white). **B** The genomic distribution of gene duplication categories. Each bar represents one gene. **C** Gene duplication plots, from left to right: all *H. schachtii* genes, *H. schachtii* genes in orthologous gene clusters categories specific to Heterodera, *H. glycines* genes in orthologous gene clusters categories specific to Heterodera, and *H. schachtii* genes in orthologous gene clusters categories specific to *H. schachtii*. Numbers above each bar represent the number of genes per category.

To understand the evolutionary origin(s) of the relatively large number of genes in *H. schachtii* in particular, and *Heterodera* spp. in general, we analysed the abundance and categories of gene duplication in the predicted exome. Compared to a related cyst nematode, *Globodera pallida* (derived using comparable methodology and of comparable contiguity) the exomes of *H. schachtii* and *H. glycines* are characterised by a relatively smaller proportion of single-copy genes (as classified by MCSanX toolkit^17^, and a relatively greater proportion of segmental duplications (at least five co-linear genes with no >25 genes between them), with relatively similar proportions of dispersed duplications (two similar genes with >20 other genes between them), proximal duplications (two similar genes with <20 other genes between them), and tandem duplications (two similar genes that are adjacent) (Fig. 2A). Genes classified as segmentally duplicated are clustered into islands (Fig. 2B) of varying gene duplication depth from 2 to 131. Setting an arbitrary duplication depth threshold of 10, approximately half of all segmentally duplicated genes (2273/4547) are grouped into 58 islands across the genome.

To understand when this large-scale segmental duplication occurred, and what genetic capital it has acted on, we cross-referenced the gene duplication analyses with a recent orthologous gene clustering of 61 species across the phylum *Nematoda*^18^. Interestingly, most (434/833) of the orthologous gene clusters that contain *H. schachtii* genes classified as segmentally duplicated also contain *H. glycines* genes classified as segmentally duplicated. We, therefore, conclude many are likely bona fide, and that at least half of the modern-day segmentally duplicated regions of the *H. schachtii* genome were present in the last common ancestor with *H. glycines*.

Interestingly, gene families that have arisen in the *Heterodera* lineage (i.e., contain more than one *H. schachtii* or *H. glycines* gene, with no representatives from all 61 other proteomes in the analysis) are numerous (1877 orthogroups, accounting for 2072 genes in *H. schachtii*) and are also enriched in segmental duplications (28% vs 17% for *H. schachtii*; Fig. 2C). Finally, *H. schachtii* specific gene families (i.e., those that only contain more than one *H. schachtii* gene, with no representatives from all 61 other proteomes in the analysis, including *H. glycines*) are also preferentially located in regions of the genome that have been segmentally duplicated (26% vs 17% of all genes; Fig. 2C). We reason that some of these segmental duplications must have arisen relatively recently (i.e., since divergence from *H. glycines*) because although it is not certain whether *H. schachtii*-specific genes arose in the *H. schachtii* lineage or were lost in the *H. glycines* lineage, if these gene families were segmentally duplicated before the divergence of *H. schachtii* and *H. glycines*, then *H. glycines* would need to have independently lost both segments to not be in the orthogroup, and this seems unlikely for all cases. Taken together, we conclude that substantial segmental duplication started in the *Heterodera* lineage after the split from *Globodera*, was still active in the *H. schachtii* lineage after the split from *H. glycines*, and has acted on old and new genetic capital.

To understand the consequences, and possible causes, of these large-scale segmental duplications, we adopted two complementary approaches: (i) a targeted approach to examine what impact this may have had on genes known to be involved in parasitism, and (ii) a non-targeted approach to examine Gene Ontology (GO) terms that are enriched in segmental duplications and/or gene families that are segmentally duplicated. We therefore additionally annotated the predicted proteome of *H. schachtii* to identify putatively secreted proteins (2669 genes, 3138 transcripts—Supplementary data 4), putative orthologues of known effectors (38 families, 209 genes, 245 transcripts—Supplementary data 5), genes putatively acquired by horizontal gene transfer (HGTs, 263 genes, 319 transcripts—Supplementary data 6), putative cell wall degrading enzymes (CWDEs, 9 families, 39 genes, 45 transcripts–Supplementary data 5), and PFAM domains (to assign Gene Ontology terms, 11,836 genes, 14,705 transcripts—Supplementary data 4).

**Fig. 3:**
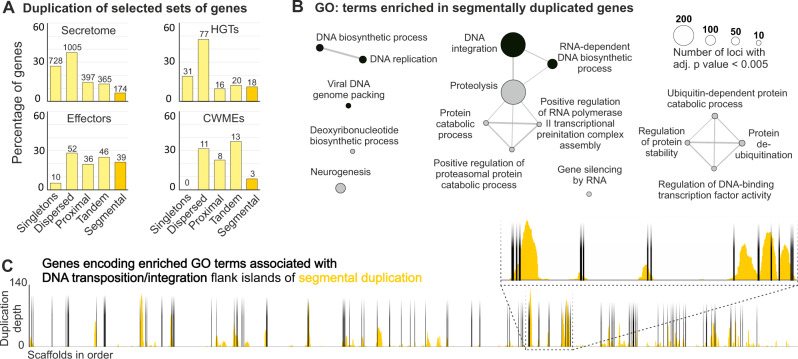
Islands of segmentally duplicated genes are flanked by genes predicted to be involved in DNA integration/transposition. **A** Gene duplication plots for all putatively secreted proteins (secretome), effectors, genes putatively acquired via Horizontal Gene Transfer (HGTs), and genes encoding Cell Wall Modifying Enzymes (CWMEs). Numbers above each bar represent to the number of genes per category. **B** GO enrichment of segmentally duplicated genes in *H. schachtii*. **C** Orange bars indicate the duplication depth of segmentally duplicated genes across the genome. Black bars (shading is vertical only and artistic, and does not carry meaning) indicate the position of genes encoding enriched GO terms associated with DNA transposition/integration in segmental duplications.

In the targeted approach, we find preferential duplication of proper subsets of the secretome and the HGTs that are directly implicated at the plant-nematode interface (i.e., effectors and CWDEs, respectively; Fig. 3A). For example, secreted proteins are not generally more duplicated than the rest of the genes in the genome, but effector genes are—including a substantial proportion in segmental duplications (~1/5th). Similarly, putative HGT events are not generally more duplicated than the rest of the genes in the genome, but the cell wall degrading enzymes are (zero single copy genes; Fig. 3A). In the non-targeted approach, we find that genes in segmental duplications are enriched in a very small set of highly-related GO terms involved in nucleic acid synthesis/manipulation and proteolysis (Fig. 3B). Many of the PFAM domains underlying these enriched annotations are associated with DNA replication/integration of viruses/transposons and, remarkably, they tend to be located towards one or both edges of islands of segmental duplication (Fig. 3C). The highly statistically significant functional enrichment of GO terms involved in DNA integration (adj. *p* value = 7.46 × 10^−92^), DNA biosynthetic process (adj. *p* value = 1.53 × 10^−11^), viral DNA genome packing (adj. *p* value = 3.86 × 10^−20^), and DNA replication (adj. *p* value = 4.67 × 10^−06^), coupled with their tendency to be positioned at the edge of segmental duplications, leads us to hypothesise that these functions may have played a role in the large-scale segmental duplication in this lineage.

### Differential expression analysis reveals contrasting evolutionary histories of host and parasite genes deployed at specific times

To understand how host and parasite genes are expressed during the parasitic life cycle, only those RNAseq reads that are uniquely mapped to the host genome, not the parasite genome, and vice versa, were used to identify host and parasite genes that were significantly differentially expressed between conditions (Log2FC > +0.5 or <−0.5 and adj. *p* value < 0.01, Supplementary data 4 and Supplementary data 7). Under these criteria, 69% (18,575) of the nematode genes, and 59% (19,071) of *A. thaliana* genes, are differentially regulated during the course of infection. To focus on the local infection-specific host response (i.e., removing changes associated with time/systemic changes), we selected only the subset of the *A. thaliana* transcriptome, 25% (8351 genes), that is differentially expressed between infected and uninfected tissues at each timepoint (hereafter referred to as differentially expressed genes). The most differentially expressed gene at each stage 10 hpi, 48 hpi, 12 dpi female, 12 dpi male, and 24 dpi female respectively) are potential marker genes for future study (Supplementary Table 3).

A majority of host and parasite differentially expressed genes (81% and 94%, respectively) were assigned to superclusters that describe different stages, or groups of stages, of the infection cycle (29 and 28 superclusters for host and parasite, respectively—e.g., Supplemental Fig. 3). For the host, the three largest clusters are of comparable size (10hpi_48hpi, 12 dpi fem._24dpi, and 10hpi_48hpi_12dpi male), and together account for over 2/5ths of all differentially expressed genes (Supplementary Table 4). For the parasite, the cluster with the most differentially expressed genes has a peak at 12 dpi males (Supplementary Table 5). It contains nearly 1/5th of all clustered differentially expressed genes, and is over twice as large as the next largest cluster (cyst). This is noteworthy because males and cysts have generally been less often considered in previous differential expression analyses in the literature compared to pre-parasitic/biotrophic stages, and yet these two life stages alone account for nearly 1/3rd of all differentially expressed genes across the life cycle. Interestingly, genes in the male-specific cluster are non-randomly distributed across the genome (for example, >31% of genes on scaffold 23 (115/364) are in the male-specific cluster, hypergeometric test, adj. *p* value = 1.51 × 10^−25^ (Supplementary Data 8)). Male-specific genes are often located in segmentally duplicated islands, even when the scaffold as a whole is not enriched, because 30% of all male-specific genes have sequence similarity to ADI82807.1 (860/2,881), and 72% of genes with sequence similarity to ADI82807.1 are in segmental duplications (2235/3094). This gene family was noted as large (474), and male-expressed, in *G. pallida*^19^. This would be an exceptionally large family of related sequences, even for a parasitic organism, and while DNA transposition may be implicated in its formation, the details of that link are at present unclear.

To analyse the evolutionary histories of clusters of genes that peak at specific life stages, we cross-referenced these data with existing analyses of orthologous gene clusters of plants and nematodes. We focused on a subset of eight plant species (*Amborella trichopoda* (outgroup), the monocots *Hordeum vulgare* and *Zea mays*, the solanaceous *Solanum lycopersicum* and *S. tuberosum*, the fabaceous *Glycine max*, and the brassicaceous *Brassica rapa* (ssp. *rapa*) and *A. thaliana* - GreenPhylDB) and a subset of eight nematodes species (the free-living nematode *Caenorhabditis elegans* (outgroup), the pine wilt nematode *Bursaphelenchus xylophilus*, the root-knot nematodes *Meloidogyne graminicola* and *M. hapla*, the potato cyst nematodes *G. pallida* and *G. rostochiensis*, and *H. glycines* and *H. schachtii*^18^). Six categories of orthologous clusters were considered for the parasite (nematodes, plant-parasites, root-knot nematodes and cyst nematodes, cyst nematodes, Heterodera, and *H. schachtii*), and six categories of orthologous clusters were considered for the plant (Magnoliopsida, Mesangiospermae (monocots and dicots), Pentapetalae, Rosids, Brassicaceae, and *A. thaliana*, Supplemental Fig. 4).

**Fig. 4:**
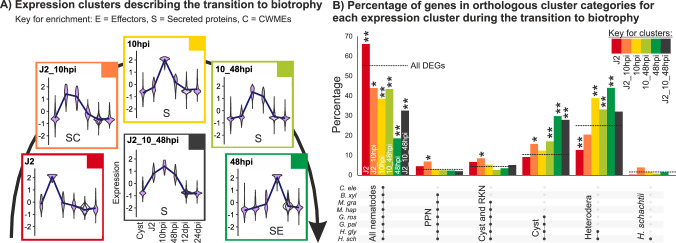
*H. schachtii* genes deployed during the transition to biotrophy are variously conserved with cyst nematodes. **A** The expression profiles of six superclusters describe the transition to biotrophy. Those clusters enriched in putatively secreted proteins (S), effectors (E), and cell wall modifying enzymes (C) are shown (e.g., SE denotes a cluster enriched in putatively secreted proteins and effectors). N.B. effectors and cell wall modifying enzymes are also putatively secreted proteins. **B** For each cluster, the proportion of genes present in each orthologous gene cluster category is shown for the following species *Caenorhabditis elegans, Bursaphelenchus xylophilus*, *Meloidogyne graminicola, M. hapla*, *Globodera pallida, G. rostochiensis*, *Heterodera glycines*, and *H. schachtii*. The dotted line represents the percentage of all DEGs in that orthologous gene cluster category for reference. Asterisks indicate empirically derived probability of randomly selecting equal-sized subsets with the same or greater differences from all DEGs (i.e., one-tailed), from 1000 iterations (*p ≤ 0.01, **p ≤ 0.001). Adjustments were not made for multiple comparisons.

We correlated the putative annotation, orthologue definition, and transcriptional clustering data to explore the relatedness and functions of subsets of differentially expressed genes. Initially, we analysed the six differential expression clusters that describe the transition to biotrophy (J2, J2_10hpi, 10 hpi, 10_48hpi, 48 hpi, and J2_10_48hpi; Fig. 4A). Of these six, five are significantly enriched in genes encoding putatively secreted proteins (hypergeometric test, false discovery rate (FDR) adj. *p* value < 0.01), the exception being the J2 specific cluster, which also contains zero known effectors. This does not mean that no effectors are expressed in the J2 stage, but rather that none are specific to the pre-infective J2 timepoint: all effectors expressed in the pre-pre-infective J2 timepoint are also expressed at some other timepoint such that they are clustered elsewhere. Importantly, at 10 hpi the nematode is still a motile J2 and, consistent with this, the only cluster significantly enriched in CWMEs is J2_10hpi (hypergeometric test, FDR adj. *p* value = 5.41 × 10^−10^) commensurate with the idea that CWMEs facilitate both entry into, but also movement within, the host. While 65% of all putative orthologues of known effectors are contained within five clusters that describe the transition to biotrophy, only one of these (48 hpi) is significantly enriched (hypergeometric test, FDR ad. *p* value = 1.08 × 10^−49^) and contains 38% of all putative orthologues of known effectors. The only other cluster enriched for effectors contains exclusively life stages involved in biotrophic interaction with the host (48hpi_12dpifem_12dpimale_24dpi, hypergeometric test, FDR adj. *p* value = 1.15 × 10^−10^).

Cross-referencing the differential expression clusters that describe the transition to biotrophy with the orthogroup analysis suggests that the transition to biotrophy is generally characterised by a proportional decrease in genes shared among nematodes, and a proportional increase in various orthologous genes cluster categories specific to subsets of plant-parasitic nematodes. On a finer scale, how the distribution of genes across the orthologous gene cluster categories compares between differential gene expression clusters that describe the transition to biotrophy is very informative. To summarise the biggest differences: the beginning of the transition to biotrophy is characterised by the greatest proportional increases in plant-parasitic nematode-specific genes and cyst and root-knot nematode-specific genes (J2_10hpi), and the greatest proportional decrease in *Heterodera*-specific genes (J2, Fig. 4B); the middle of the transition (10 hpi) is characterised by a proportional increase in *Heterodera*-specific genes; and the end of the transition (10hpi_48hpi and to a greater extent 48 hpi) is characterised by a proportional increase in cyst nematode-specific genes and *Heterodera*-specific genes. Considering the functional annotation of genes in these clusters (Fig. 4A), we conclude that genes expressed exclusively in the pre-infective J2 are not involved in host manipulation (unless they are also expressed in another timepoint (e.g., J2_10hpi)). Given that the 10 hpi cluster is characterised by a larger proportional increase in *Heterodera*-specific genes than cyst nematode-specific genes, coupled with the fact that the greatest difference between infected and uninfected plant tissue is at 10 hpi and dominated by immunity-related functions, these data appear consistent with the idea that a greater degree of specialisation from the parasite is needed to overcome the early, immunity-related, host response. Finally, we conclude that *H. schachtii* is a model for the most agriculturally important cyst nematode, *H. glycines*, because *H. schachtii*-specific genes represent a tiny proportion of those expressed during the transition to biotrophy (generally <5%).

**Fig. 5:**
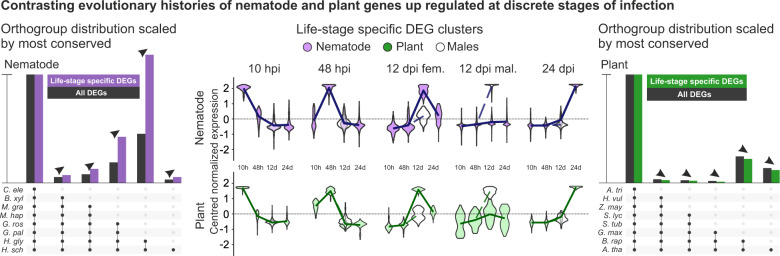
Contrasting evolutionary histories of host and parasite genes deployed at specific times of infection. Differential expression superclusters that describe discrete stages of infection (centre) for either the host (closed green) or the parasite (closed purple) where hpi = hours post infection and dpi = days post infection. Open violins represent males, or plant tissue associated with males. The orthogroup distributions (scaled to the most conserved category) are shown for eight nematode species (left - *Caenorhabditis elegans, Bursaphelenchus xylophilus*, *Meloidogyne graminicola, M. hapla*, *Globodera pallida, G. rostochiensis*, *Heterodera glycines*, and *H. schachtii*) and eight plant species (right - *Amborella trichopoda, Hordeum vulgare*, *Zea mays*, *Solanum lycopersicum, S. tuberosum*, *Glycine max*, *Brassica rapa* (ssp. *rapa*) and *A. thaliana*). Arrowheads indicate a proportional increase or decrease compared to all differentially expressed genes (DEGs, black). The probability <0.05 was empirically derived (from 1000 random samples of equal-sized subsets) for all five categories with a proportional increase (in the case of the nematode) or a proportional decrease (in the case of the plant) compared to all DEGs (i.e., one-tailed). Adjustments were not made for multiple comparisons.

Subsequently, we analysed the five differential expression clusters, of the host and of the parasite, that describe discrete stages of infection (10 hpi, 48 hpi, 12dpi_male, 12dpi_female, and 24dpi_female; Fig. 5). Strikingly, host and parasite genes upregulated at the same time of infection have different, and contrasting, evolutionary histories within their respective phyla. Parasite genes in expression clusters that peak at individual times of infection are generally less well conserved across the phylum Nematoda when compared to all differentially expressed genes in the experiment (Fig. 5 and Supplemental Fig. 5). In contrast, host genes in expression clusters peak at those same times of infection are generally better conserved across the kingdom Plantae when compared to all differentially expressed genes in the experiment (Fig. 5 and Supplemental Fig. 5). Although these evolutionary time scales are not directly comparable, these data may suggest that infection-specific gene expression is characterised by lineage-specific nematode genes (including, for example, effectors) modulating the expression of widely conserved plant genes (including, for example, genes involved in the development of the feeding site).

### Congruent differential expression of metabolic pathways during infection highlights nematode susceptibility genes

To understand the roles of widely conserved host genes, and lineage-specific parasite genes, that are differentially regulated during biotrophy (Fig. 5), we used the KEGG Automatic Annotation Server Ver. 2.1 (https://www.genome.jp/kaas-bin/kaas_main) to annotate metabolic pathways in the host (3,860 KO terms on 9,240 genes) and the parasite (3479 KO terms on 4990 genes (Supplementary Data 9)). Given the partial overlap of KO terms (1,998 in common between host and parasite) we examined pathways that were incomplete in the parasite, but complemented by the host.

**Fig. 6:**
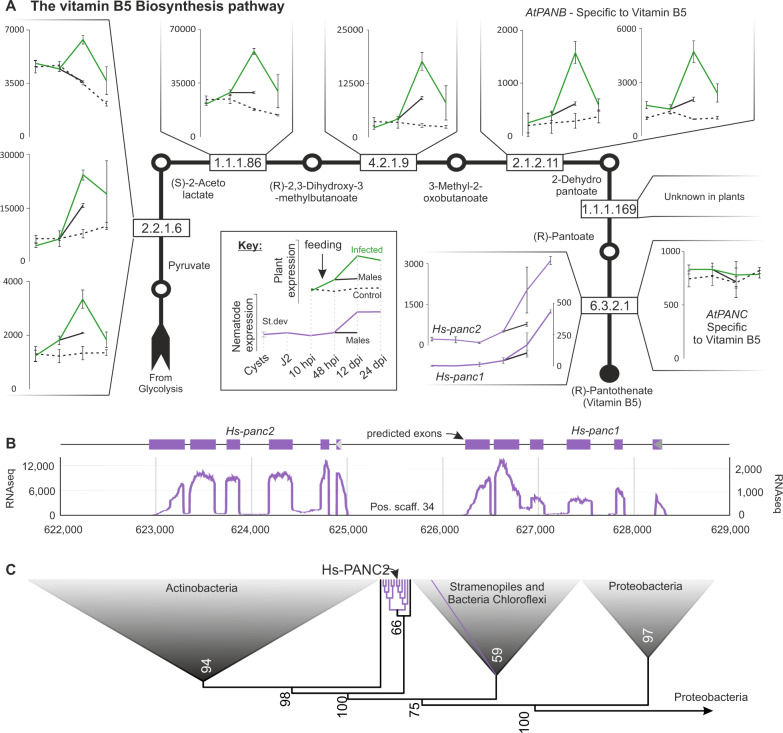
Congruent differential expression of the vitamin B5 biosynthesis pathway between kingdoms is enabled by a horizontal gene transfer from bacteria. **A** The linear pathway of vitamin B5 biosynthesis. Products/substrates are indicated with circles, enzymatic reactions (and corresponding Enzyme Commission (EC) codes) with squares. For each species, the expression profiles of the gene/s annotated with the EC codes at each step are shown (green for host, purple for parasite), if present. Error bars indicate the standard deviation of the mean (centre point, *n* = 3 biologically independent samples). **B** The two predicted *H. schachtii panc* orthologues are adjacent, in the same orientation, and located towards the middle of a 1.3 Mb scaffold. RNAseq read mapping (graph) and intron:exon structure of both genes (5:6, blocks above) are similar, and confirm that they do not arise from contamination. **C** A subset of the phylogenetic tree inferred from the protein alignment of the top 50 most similar sequences identified in the NCBI non-redundant database to each of 14 animal putative *PANC* (the full phylogeny is available in Supplemental Fig. 6). Most PANC homologues from plant-parasitic nematodes (purple), including Hs-PANC2, group in a single monophyletic sub-clade with sequences from actinobacteria.

The vitamin B5 (pantothenate) biosynthetic pathway (M00119) is an example of congruent differential expression of metabolism involving widely conserved host genes and lineage-specific parasite genes. The complete pathway is conserved in most plants, and all steps (even when more than one gene encodes a particular function) are upregulated during infection of *A. thaliana* with *H. schachtii* (Fig. 6A), with the exception of the last step pantothenate synthetase (*AtPANC*). The dominant peak of expression in all upregulated cases is at 12 dpi in the syncytia associated with the female. The expression of this pathway in syncytia associated with the 12 dpi male is either indistinguishable from the control (e.g., 2.2.1.6), or significantly upregulated compared to the control, but much less highly upregulated than in syncytia associated with the 12 dpi female and approximately at the same level as in the syncytia associated with the 24 dpi female. While all animals require vitamin B5 in order to complete the basal metabolism of proteins, carbohydrates, and lipids, the biosynthesis pathway is considered absent (vitamin B5 is an essential nutrient for animals). Unusually for an animal, the *H. schachtii* genome encodes two genes (*Hsc_gene_23032* and *Hsc_gene_23033*) annotated to carry out only the last step (PANC) of the vitamin B5 biosynthesis pathway^20^. These putative *H. schachtii* PANC-encoding genes (*Hs-panc*) are dissimilar in sequence to plant orthologues. Querying the non-redundant nucleotide archive, the most similar sequences to *Hs-panc* are from a few animals with obligate interactions with plants (primarily, although not exclusively, plant-parasitic nematodes) and the vast majority from bacteria (Fig. 6C). Phylogenetic analyses group the *Hs-panc*, along with many other plant-parasitic *panc*-like sequences, towards the base of a well-supported clade (100 bootstrap) containing sequences primarily from actinobacteria (the most closely clustered sequence being from *Candidatus Rhodoluna planktonica*, WP_070954084.1 [https://www.ncbi.nlm.nih.gov/protein/WP_070954084.1?report=genpept], a well characterised but as-yet uncultured organism). Importantly, both *Hs-panc* encode several well-supported introns and are in the middle of a large 1.3 Mb scaffold surrounded by classical nematode genes, disproving the possibility of contamination (Fig. 6B). The most parsimonious explanation for the existence of *panc* in the *H. schachtii* genome is, therefore, their acquisition by horizontal gene transfer from bacteria to the last common ancestor of the suborder Hoplolaimina—indicative of an important role in plant-nematode interactions across the group - and subsequent tandem duplication in *H. schachtii* or its progenitor. Remarkably, *Hs-panc* complement the upregulation of the first steps in the pathway in the host (and the absence of upregulation of the last step in the pathway in the host): *Hs-pancs* are upregulated in the 12 dpi female, to a lesser extent in the 12 dpi male, and to a greater extent in the 24 dpi female (Fig. 6A).

Taken together, we conclude that the vitamin B5 (pantothenate) biosynthetic pathway is congruently differentially expressed between kingdoms in this interaction. The presence of *panc*-like sequences in two other groups of animals outside the plant-parasitic nematodes (*Tetranychus urticae* - a species of plant-feeding mite generally considered to be a pest—XM_015927639.1^21^, and *Apis mellifera*—the western honey-bee—XM_001123024.1 is intriguing given their similarly obligate relationship with plants (Supplemental Fig. 6). In the case of *A. mellifera*, it is somewhat more ambiguous than for the plant-parasitic nematodes and the mite. The predicted protein sequences of the *A. mellifera* PANC are 65% identical with the most similar bacterial sequence in NCBI (with much less similar nucleotide sequences), the gene does not contain introns, however, and it is on a short scaffold. Other examples of congruently differentially expressed metabolic pathways that warrant further investigation are more complicated (due to not being exclusively linear pathways) but show similar trends (e.g. biotin (Supplemental Fig. 7 and Supplementary Tables 6 and 7 for parasite and host respectively), riboflavin, stilbenoid, and vitamin B6).

**Fig. 7:**
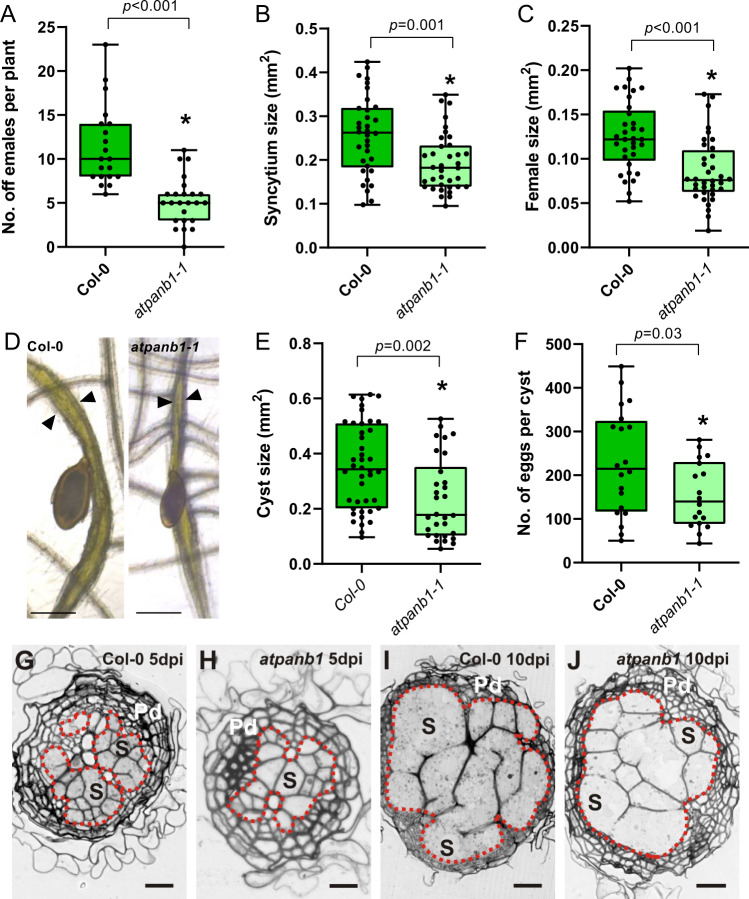
*AtPANB1* is required for cyst nematode parasitism. **A** Number of female cyst nematodes present per plant root system at 14 dpi (Col-0, *n* = 20; *atpanb1-1*, *n* = 24). **B** Size of syncytia at 14 dpi. Female-associated syncytia were randomly selected and their outlines were measured (Col-0, *n* = 33; *atpanb1-1*, *n* = 36). **C** Size of female nematodes at 14 dpi. Female cyst nematodes were randomly selected and their outlines were measured (Col-0, *n* = 33; *atpanb1-1*, *n* = 36). **D** Example images of nematode infection on Col-0 and *atpanb1-1* mutants. Arrowheads indicate syncytium boundaries and asterisks indicate female nematodes. **E** Size of cysts at 42 dpi. Nematode cysts were randomly selected and their outlines were measured (Col-0, *n* = 43; *atpanb1-1*, *n* = 32). **F** Number of eggs per cyst at 42 dpi. Cysts were randomly selected and their egg numbers were counted (Col-0, *n* = 20; *atpanb1-1*, *n* = 19). **A**–**C**, **E**, **F**). Experiments were performed 3–8 times independently with a similar outcome. Data from one experiment is shown. Data were analysed using Student’s *t* test (two-sided; *α* = 0.05) and asterisks indicate significantly different means. For box plots, centre line is median; box limits are upper and lower quartiles; whiskers are minimum and maximum value; points are all individual values superimposed on graphs. Source data are provided as a source data file. **G**–**J** Light microscopy images of cross sections taken through the widest part of syncytia induced by *H. schachtii* in Col-0 (**G**, **I**) and *atpanb1-1* (**H**, **J**) roots at 5 (**G**, **H**) and 10 dpi (**I**, **J**). Syncytial elements and neighbouring enlarged cells presumably undergoing transition into syncytial elements are outlined with red dotted lines. Pd periderm, S example (not all) cells contributing to the syncytium. Scale bars: 20 µm.

**Fig. 8:**
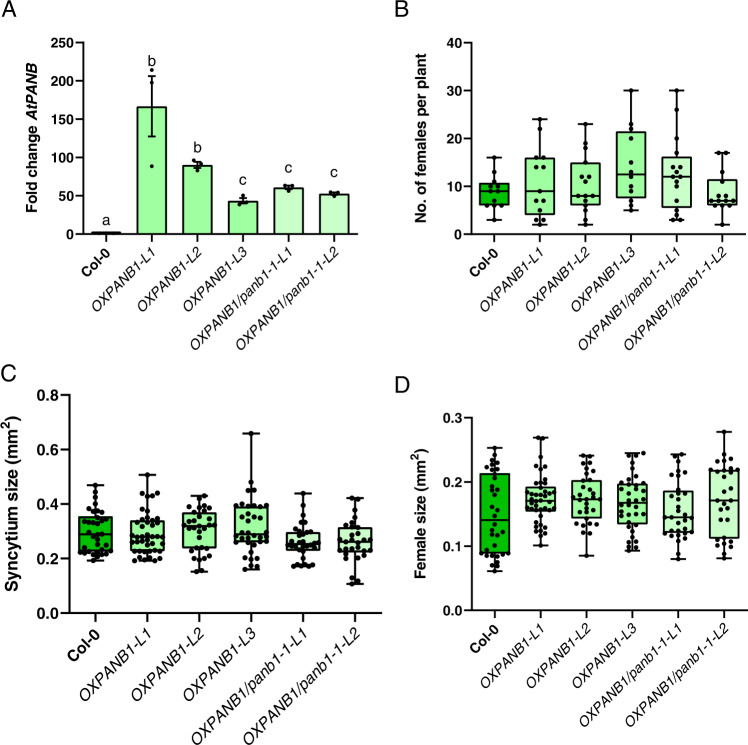
Overexpression of *AtPANB1* restores susceptibility to cyst nematodes. **A** Quantitative reverse transcription-polymerase chain reaction (qRT-PCR) confirmation of the increase in *AtPANB1* transcript in overexpression lines of the Col-0 (*OXPANB1* lines L1, L2, L3) or *atpanb1-1* (*OXPANB1/panb1-1* lines L1 and L2) background. Bars represent mean ± SE. Data were analysed using a one-way analysis of variance (ANOVA) followed by Tukey’s HSD post hoc test (*α* = 0.05). Experiments were repeated three times independently (*n* = 3) and different letters indicate significantly different means. *AtPANB1* transcript levels were significantly increased in comparison to all tested lines (*p* < 0.001). **B** Number of female cyst nematodes present per plant root system at 14 dpi (Col-0, *n* = 12; *OXPANB1-L1*, *n* = 13; *OXPANB1-L2, n* = 15; *OXPANB1-L3, n* = 12; *OXPANB1/panb1-1-L1*, *n* = 16; *OXPANB1/panb1-1-L2*, *n* = 14). **C** Size of syncytia at 14 dpi. Female-associated syncytia were randomly selected and their outlines were measured (Col-0, *n* = 34; *OXPANB1-L1*, *n* = 42; *OXPANB1-L2, n* = 31; *OXPANB1-L3, n* = 36; *OXPANB1/panb1-1-L1*, *n* = 32; *OXPANB1/panb1-1-L2*, *n* = 29). **D** Size of female nematodes at 14 dpi. Female nematodes were randomly selected and their outlines were measured (Col-0, *n* = 36; *OXPANB1-L1*, *n* = 44; *OXPANB1-L2, n* = 33; *OXPANB1-L3, n* = 38; *OXPANB1/panb1-1-L1*, *n* = 34; *OXPANB1/panb1-1-L2*, *n* = 31). **B**–**D** Experiments were performed 3–6 times independently with a similar outcome. Data from one experiment is shown. Source data are provided as a source data file. Data were analysed using a one-way analysis of variance (ANOVA) followed by Tukey’s HSD post hoc test (*α* = 0.05). No significant difference (n.s.) was detected. For box plots, centre line is the median; box limits are upper and lower quartiles; whiskers are minimum and maximum value; points are all individual values superimposed on graphs.

-These data support the hypothesis that the plant-encoded enzymes of the first part of the vitamin B5 biosynthesis pathway support nematode infection, and may thereby function as nematode susceptibility genes. To investigate whether *AtPANB1* and *AtPANB2* (the penultimate step in the pathway, and the last step upregulated upon infection) plays a role in cyst nematode parasitism, we ordered T-DNA insertion lines for both *AtPANB1* and *AtPANB2*. After several unsuccessful attempts to generate a homozygous loss-of-function for *AtPANB2*, we focused our further analysis on *AtPANB1* (*atpanb1-1*; Supplemental Fig. 8). With the exception of a slight delay in flowering time, *atpanb1-1* was phenotypically indistinguishable from wild-type in the absence of infection: hypocotyl length, root and shoot fresh weight, and siliques sizes, were not altered compared to Col-0 (Supplemental Fig. 9). No morphological differences were observed in roots between Col-0 and *atpanb1-1*. During infection, however, the following marked differences were noted between the mutant and wild-type lines. We found that *atpanb1-1* supported fewer females at 14 dpi (Fig. 7A; *p* value < 0.0001). Moreover, even those nematodes that were able to infect the mutant are less fit as evidenced by the formation of smaller syncytia (Fig. 7B, D, *p* value < 0.0001), smaller females (Fig. 7C, D, *p* value = 0.02), smaller cysts (Fig. 7E; *p* value = 0.049), and lower average number of eggs per cyst (Fig. 7F; *p* value = 0.049). We have not noted striking differences in egg morphology, size or hatching rate. However, we have not quantified these parameters. Importantly, the reduced susceptibility of *atpanb1-1* to nematodes can be rescued by overexpressing *AtPANB1* under the control of a *35* *S* promoter in the *atpanb1-1* background (Fig. 8). These experiments were performed multiple times (at least three) independently, with the same outcome (Data for additional replicates are available in the Source Data file)

Next, we conducted microscopic examinations of anatomical and ultrastructural features of syncytia induced in roots of *atpanb1-1* plants and compared them with syncytia induced in wild-type Col-0 plants (Fig. 7G–J and Supplemental Fig. 10). In both genotypes, syncytia were induced in the vascular cylinder and they were composed of vascular cylinder cells only (Fig. 7G–J)^9^. In wild-type Col-0, syncytia were surrounded by a continuous layer of periderm (Fig. 7G; Supplemental Fig. 10B, D)^22^, excluding regions next to nematode heads (Supplemental Fig. 10A, C). However, in the case of syncytia induced in *atpanb1-1* roots, the periderm layer was not yet differentiated next to the nematode head (Supplemental Fig. 10E) and syncytia developed poorly at 5 dpi (Fig. 7H and Supplemental Fig. 10F). Overall, syncytia induced in *atpanb1-1* were less well developed than in Col-0 plants as indicated by reduced size and number of cells incorporated into syncytia. This was especially evident at 5 dpi (Fig. 7G, H; Supplemental Fig. 10A, B, E, F). At 10 dpi, syncytia induced in *atpanb1* roots were still smaller on cross sections than syncytia induced in wild-type plants, but this difference was less apparent (Fig. 7I, J and Supplemental Fig. 10D, H). At the ultrastructural level, the organisation of syncytial protoplasts in Col-0 plants was typical for syncytia induced in Arabidopsis roots. Syncytial cytoplasm is marked by a high density of organelles (Supplemental Fig. 10I, J)^9, 10^. A contrasting situation was found in syncytia induced in *atpanb1-1* roots (Supplemental Fig. 10K, L). At the widest parts of syncytia, some distance from the nematode heads, the syncytial cytoplasm exhibits large regions almost free from plastids, mitochondria and structures of endoplasmic reticulum both at 5 and 10 dpi. Syncytia are metabolically hyperactive sinks from which nematodes feed throughout their life cycle. The ultrastructural features developed in *atpanb1-1* are unusual, and might indicate that syncytia induced in these plants are metabolically less active and therefore less efficient in supporting nematode development leading to/stemming from the formation of smaller and undernourished females. These data suggest that a reduction in pantoate supply in *atpanb1-1* mutant compromises nematodes’ ability to synthesise vitamin B5, leading to smaller females, which are unable to fully support the establishment and/or maintenance of a syncytium. It is therefore likely that the syncytial phenotype observed is an indirect effect caused by the nematode’s general lack of fitness.

To investigate whether *AtPANB1* plays a similar role in other plant-interacting organisms, we analysed changes in susceptibility of *atpanb1-1* towards the necrotrophic fungus *Botrytis cinerea*, and the beneficial endophyte *Serendipita indica*. In both cases, no differences were detected between *atpanb1-1* and the wild-type control (Supplemental Fig. 11A, B). Next, we investigated whether the decrease in susceptibility of *atpanb1-1* to cyst nematodes is due to changes in classical immune responses. To this end, we tested ROS production, a hallmark of basal defence in plants, upon treatment of an immunogenic peptide, flg22. We did not observe any changes in ROS burst, both over time or cumulative (Supplemental Fig. 11C). Taken together, these data suggest that *AtPANB1*, the last step in the pathway upregulated during infection, is a specific non-immunity-related nematode susceptibility gene. Given that this pathway is linear, and that all previous steps are similarly upregulated, the implication is a series of nematode susceptibility genes for future study. The wide conservation of *panc* in plant-parasitic nematodes (Fig. 6B), and where we have data on their similar upregulation during parasitism (e.g., *G. rostochiensis*, *Gr-panc*—GROS_g05752—Supplemental Fig. 12), extends this intriguing possibility to at least the cyst nematodes, but perhaps most other plant-parasitic nematodes of global agricultural importance^23^.

Balancing the cost vs reward of any S gene is an important consideration in the application of the technology. Generally, one would aim to achieve a partial knockout of function such that infection is impaired, but the useful biology of the plant is not impaired. Intuitively one might think that the gene with the highest expression is the most suitable for knockout—in the case of S genes, weak phenotypes (with no or minimal impact on “normal” plant physiology, but large impacts on parasitic physiology, as shown herein) are ideal. Redundancy in the pathway (as is the case for *AtPANB*) can help in this regard, and is anticipated to be particularly relevant to plant-parasitic nematodes given the extreme metabolic strain and relative fragility of the feeding site. With the exception of a slight delay in flowering time, *atpanb1-1* was phenotypically indistinguishable from wild-type in the absence of infection. Generating a CRISPR-based allelic series of panb mutants in one of the major crop plants infected by cyst nematodes, for example, potato, would be an excellent use case because: the pathogen is closely related; the crop is polyploid (and so a wide variety of reduction of function mutants can be generated); flowering time is not required for crop production (and may not be similarly affected); and finally the corresponding homologue of HsPANC (GROS_g05752) is similarly and almost exclusively upregulated during biotrophy (14 days post-infection, Supplemental Fig. 12).

### Trans-kingdom compartmentalisation of the vitamin B5 pathway

**Fig. 9:**
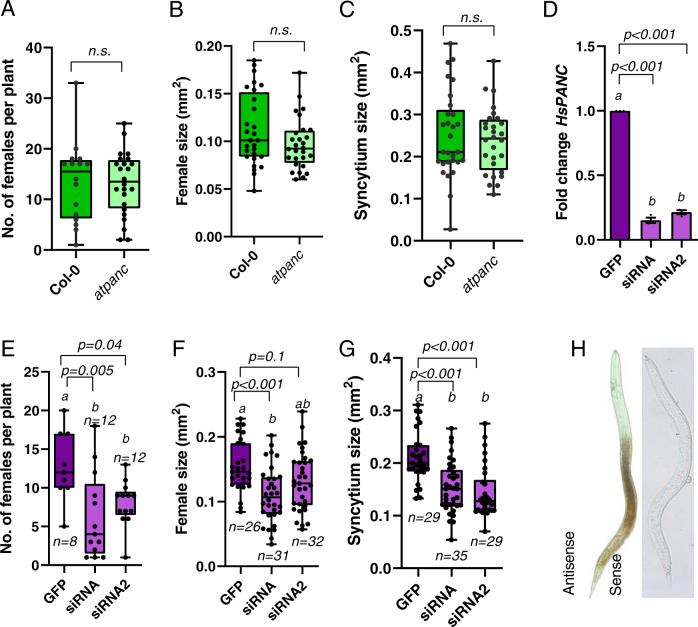
*Hspanc*, but not *AtPANC*, is required for cyst nematode parasitism. **A** Number of female cyst nematodes present per plant root system at 14 dpi (Col-0, *n* = 16; *atpanc*, *n* = 24). **B** Size of syncytia at 14 dpi. Female-associated syncytia were randomly selected and their outlines were measured (Col-0, *n* = 28; *atpanc*, *n* = 28). **C** Size of females at 14 dpi. Female nematodes were randomly selected and their outlines were measured (Col-0, *n* = 28; *atpanc*, *n* = 28). **A**–**C** Experiments were performed three times independently with a similar outcome. Data from one experiment is shown. Source data are provided as a source data file. Data were analysed using Student’s *t* test (two-sided; *α* = 0.05). No significant difference (n.s.) was detected. **D** Change in transcript abundance of *HsPANC* gene in J2 nematodes soaked in siRNA targeting *HsPANC* or GFP. Bars represent mean ± SE. Data were analysed using a one-way analysis of variance (ANOVA) followed by Tukey’s HSD post hoc test (*α* = 0.05) and different letters indicate significantly different means. Experiments were repeated three times independently (*n* = 3). **E** Number of females present per root system at 14 dpi (GFP, *n* = 9; siRNA1, *n* = 13; siRNA2 = 13). **F** Size of females at 14 dpi. Female nematodes were randomly selected and their outlines were measured (GFP, *n* = 27; siRNA1, *n* = 32; siRNA2 = 33). **G** Size of syncytia at 14 dpi. Female-associated syncytia were randomly selected and their outlines were measured (GFP, *n* = 30; siRNA1, *n* = 36; siRNA2 = 36). **E**–**G** Experiments were performed three times independently with a similar outcome. Data from one experiment is shown. Source data are provided as a source data file. Data were analysed using a one-way analysis of variance (ANOVA) followed by Tukey’s HSD post hoc test (*α* = 0.05) and different letters indicate significantly different means. (**A**–**C** and **E**–**G**) Centre line is median; box limits are upper and lower quartiles; whiskers are minimum and maximum value; points are all individual values superimposed on graphs. **H**) In situ hybridisation of the *HsPANC* gene in J2 of cyst nematode.

In contrast to *AtPANB*, *AtPANC* is neither upregulated in syncytium nor plays a role in cyst nematode infection: loss-of-function homozygous mutants for *AtPANC* (Fig. 6; Supplemental Figs. 6 and 7) are indistinguishable from wild-type plants during infection (Fig. 9A–C). Complementing this lack of upregulation, the expression of both *Hs-panc1*, but particularly *Hs-panc2*, is not only increased after onset of parasitism (48 hpi) but remains high as the nematode proceeds through the rest of its life cycle, pointing to a functional role for *Hs-panc*, in particular *Hs-panc2*, in parasitism (Fig. 6A). To test this hypothesis experimentally, we used in vitro RNAi targeting the more highly expressed paralogue (by a factor of nearly 10) *Hs-panc2* (Supplemental Fig. 13), which caused a significant decrease in expression of *Hs-panc2* (Fig. 9D) and resulted in fewer female nematodes infecting wild-type Col-0 (Fig. 9E). The average sizes of the females and syncytia were also significantly reduced on Col-0 infected with nematodes showing a reduced expression of *Hs-panc* (Fig. 9F, G). Inconsistent effects were seen on the number of males (Supplemental Fig. 14). Pre-infective juveniles of cyst nematodes (pre-J2s) are non-feeding, essentially arrested in development, and use their lipid reserves to locate and invade the host plants. They start feeding only after their feeding sites are established. It is therefore highly unlikely that knocking down *HsPANC* affects nematode behaviour pre-infection (indeed, *HsPANC* is lowly expressed when the nematode is outside the plant). Decreased expression of *HsPANC* is hypothesised to compromise the fitness of nematodes post infection, which will in turn affect nematode’s ability to establish and/or maintain their feeding sites, and therefore manifest in the decreased infection measured herein.

Importantly, *Hs-panc* functions in the nematode. *Hs-panc* does not encode an N-terminal secretion signal, and in situ hybridisation of digoxigenin-labelled probes to *Hs-panc* mRNA reveals expression throughout the nematode body with a particularly strong signal in the intestinal region of the nematode (Fig. 9H). Taken together, we conclude that the contribution of *Hs-panc* to parasitism success is due to its role in the nematode, during parasitism.

**Fig. 10:**
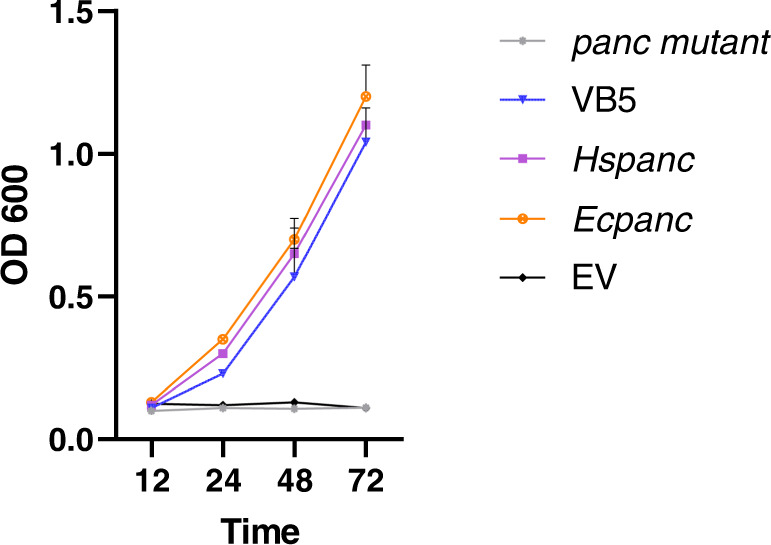
*Hspanc* encodes a functional PANC. Functional complementation of *panc*-deficient *E. coli* strain (AT1371) (*panc* mutant) containing the empty plasmid (EV), exogenous pantothenate (VB5), nematode panc (*Hs-panc*), and the bacterial panc (*Ec-panc*). Lines represent mean ± SE from three independent experiments (*n* = 3). Source data are provided as a source data file.

To understand how *Hs-panc* contributes to parasitism success in the nematode, we tested whether *Hs-pancs* encode a functional enzyme. *Hs-panc1* cDNA was cloned into PUC18, and was tested for its ability to complement *panc* mutant from *Escherichia coli* (AT1371). The *E. coli panc* gene served as a positive control. All strains grew well in the presence of pantothenate in the growth medium. In absence of exogenous pantothenate, the *panc* mutant containing the empty vector did not grow. However, the strains transformed with either bacterial or nematode *panc* grew well with no significant differences detected between strains (Fig. 10). We, therefore, concluded that *Hs-panc* encodes a bona fide PANC.

The presence of *panc* homologues in the cyst nematode’s genome indicates an evolutionary benefit, but the molecular fundamentals of this peculiar separation remain elusive. Previous studies show that plant PANC is subjected to uncompetitive substrate inhibition at higher pantoate concentrations^24^. Similarly, the activity of plant PANC is also inhibited by products, and in particular by the accumulation of pantothenate^25^. *E. coli*, on the other hand, produces vitamin B5 in large amounts, and any excess is secreted into the medium, strongly suggesting that vitamin B5 biosynthesis, including PANC activity, is not very tightly regulated. In comparison to bacteria, plants are unlikely to depend on continuous excretion of vitamin B5, as this would require an elaborate transport system^26^. The biosynthesis of vitamin B5 is therefore expected to be more tightly regulated in plants. In line with this idea, PANC from plants e.g., *Lotus japonicus* is shown to have strong substrate inhibition by pantoate, whereas enzymes from *E. coli* and *Mycobacterium tuberculosis* follow hyperbolic kinetics^24, 27, 28^. Similarly, a comparative analysis of kinetic properties showed that although the two-step reaction mechanism is conserved between the AtPANC and EcPANC, AtPANC exhibits large allosteric effects, whereas EcPANC displays essentially non-allosteric behaviour^26^. Based on these observations, we propose a hypothesis (Supplemental Fig. 15) that compartmentalisation of the vitamin B5 pathway between plants and nematodes helps to avoid feedback/feed-forward inhibition and ensures a consistent supply of vitamin B5 to rapidly developing nematodes. Clarifying further details of the nematode vitamin metabolism, including details of the activity of HsPANC and AtPANC may aid in the development of nematode-resistant germplasm through the targeted deletion of susceptibility genes.

To conclude, we have shown that *H. schachtii* has the largest genome, and second-largest exome, of any cyst nematode analysed to date. Substantial segmental duplication contributed to the large gene number, it started at some point in the Heterodera lineage after the split from Globodera, was still active in the *H. schachtii* lineage after the split from *H. glycines*, and has acted on old and new genetic capital. A reference trans-kingdom, lifestage-specific, transcriptome of *H. schachtii* and *A. thaliana* reveals that host and parasite genes upregulated at the same time of infection have different, and contrasting, evolutionary histories within their respective phyla. Nevertheless, congruent differential expression analysis of metabolic pathways in this time course transcriptome highlights nematode susceptibility genes involved in vitamin B5 biosynthesis. Taken together, we conclude that the vitamin b5 biosynthesis pathway of the plant-nematode hologenome starts in the plant, ends in the nematode, and is putatively enabled by a horizontal gene transfer from a bacterium.

## Methods

In all cases, measurements were taken from distinct samples.

### Flow cytometry

Approximately 50 μl of compacted live *H. schachtii* J2s (Bonn population) in a 1.5 ml Eppendorf tube were resuspended in 10 μl of extraction buffer (PBS with 0.01% Triton X) and disrupted with a micro pestle. Lysed nematodes were resuspended in 500 μl of extraction buffer and the debris was filtered through 100 μm and then 30 μm filters (CellTrics). Up to 250 μl of the filtrate was added to 1 ml of staining buffer (100 mM Tris (pH 7.4), 150 mM NaCl, 1 mM CaCl_2_, 0.5 mM MgCl_2_, 2 μg ml^−1^ propidium iodide, and 0.18 mg ml^−1^ RNase A) and incubated on ice in the dark for 1 h. Stained nuclei were counted on an Accuri C6 flow cytometer (BD Biosciences-USA). For *A. thaliana*, 1–2 leaves were removed and placed on a Petri dish, immersed in 250–500 μl of extraction buffer. The leaf tissue was finely cut with a razor blade. The remaining liquid and leaf debris were filtered, stained, and counted as nematode samples. Flow cytometry data were analysed with FlowJo.

### Trans-kingdom transcriptome sampling, sequencing, and analysis

Seeds of *A. thaliana* ecotype Columbia-0 (Col-0 - the same were used for all experiments unless otherwise stated) were surface-sterilised by washing in 70% (v/v) ethanol for 5 min followed by washing with 2% (w/v) sodium hypochlorite for 3 min. Seeds were rinsed twice with sterile water and were dried on sterile Whatman filter paper before planting. Plants were grown in Petri dishes in agar supplemented with modified Knop’s nutrient medium at 23 °C under long-day conditions with 16 h light and 8 h darkness. Second-stage juveniles were harvested from nematode stock culture, which has been maintained on mustard roots under sterile conditions for 20 years. Twelve-day-old plants were inoculated with 60–70 surface-sterilised juveniles. Small root segments containing nematodes were marked under a stereo microscope. The infected area including nematodes was then hand-dissected and transferred to liquid nitrogen, avoiding confounding structures such as lateral roots and root tips. Adjacent (basipetal or acropetal) uninfected root segments, similarly avoiding confounding structures, were collected as control tissue in order to better identify the local differences in gene expression during infection (Supplemental Fig. 16). Several hundred such segments were collected for each biological replicate.

Frozen tissue was lysed to a fine powder with a tissue lyser at 30 Hz (2 × 2 min). RNA was extracted from frozen powder using the RNeasy Plant Mini Kit (Qiagen) following the manufacturer’s instructions and using both the optional shredder columns and on-column DNA digestion. RNA quality was assessed using Bioanalyzer. Library construction (150 bp paired-end stranded RNAseq) and sequencing was carried out by Novogene (Bioproject: PRJNA722882 [https://www.ncbi.nlm.nih.gov/nuccore/2062503727]). Additional sequencing of the early mixed time points (10 h and 48 h post infection) was carried out to provide enough coverage of the nematode exome (Fig. 1).

### Genome extraction, sequencing, and assembly

High molecular weight DNA was extracted from pools of approximately one million frozen *H. schachtii* J2s (Bonn population - the same were used for all experiments unless otherwise stated) using the phenol/chloroform/isoamyl alcohol extraction method. In brief: pelleted juveniles (in a 1.5 ml eppendorf) were resuspended in 160 μl of extraction buffer (0.1 M Tris (pH 8.0), 0.5 M NaCl, 50 mM EDTA, 1% (w/v) SDS). The pellet was ground using a micro pestle for less than 30 s and until the pellet thawed, and 40 μl of proteinase K (20 mg ml^−1^) was added. The samples were incubated at 55 °C for 24 h, mixing gently but often. RNase A (10 μl of 10 mg ml^−1^) was added, incubated at room temperature for 10 min, and mixed with equal volumes of phenol/chloroform/isoamyl alcohol solution (25:24:1). Phenol/chloroform extraction, chloroform back extraction, and ethanol precipitation was performed according to the PacBio protocol (https://www.pacb.com/wp-content/uploads/2015/09/SharedProtocol-Extracting-DNA-usinig-Phenol-Chloroform.pdf).

Two PacBio libraries were prepared from extracted DNA. First, a 25 kb library (DNA was fragmented to 25 kb, size selection of the final library was performed using BluePippin at 10 kb cutoff), and subsequently a 35 kb library (DNA was fragmented to 35 kb, size selection was performed with BluePippin at 15 kb cutoff). Each library was sequenced on Pacific Biosciences Sequel instrument using Sequel Polymerase v2.1, SMRT cells v2 LR and Sequencing chemistry v2.1. Loading was performed by diffusion, in total 4 SMRT cells were used (movie time: 1200 min). Sequencing was provided by the Norwegian Sequencing Centre (www.sequencing.uio.no), a national technology platform hosted by the University of Oslo and supported by the Functional Genomics and Infrastructure programmes of the Research Council of Norway and the Southeastern Regional Health Authorities. In addition, one 150 bp paired-end (350 bp insert size) Illumina library was prepared from the same DNA (NEBNext Ultra II DNA Library Prep Kit for Illumina) and sequenced to ~140 fold coverage (24.6 Gb) using the service provided by NovoGene (GenBank Accession PRJNA722882 [https://www.ncbi.nlm.nih.gov/nuccore/2062503727]).

All PacBio subreads (Bioproject: PRJNA722882) were error corrected using Canu snapshot v1.7 + 137 changes (r8829 73d5caa1b1087b65f7853ecbebc1bb1dcbd1bc14)^29^, with the following additional parameters: corOutCoverage = 300 corMhapSensitivity=normal. This allowed all the PacBio data to be used in the error correction phase instead of 40×, resulting in 1,013,782 corrected reads. Corrected reads were assembled using wtdbg2 Version: 2.1 − 20181007^30^. A variety of parameters were tested, and each assembly was assessed for assembly size, BUSCO^31^ version 1.1b using Eukaryota data set for completeness score (pre-trained on *G. pallida* Augustus version 3.2.1 models^32^), and RNAseq mapping STAR^33^. The final assembly used the following parameters: -L 5000 -p 19 -AS2. CEGMA^34^ was also used to quantify the completeness of assemblies based on a set of core eukaryotic genes.

The assembly was subjected to BlobTools version 1.0 analysis^35^ to identify and remove putative contaminant contigs. Briefly, reads were mapped to the assembly using minialign version 0.5.2 (https://github.com/ocxtal/minialign) to determine the coverage of the assembled contigs. The contigs were compared to the GenBank nt database using BLASTn (reporting taxonomic information). Contigs were then classified based on the weight of the BLAST hits. Contigs identified as fungal, bacterial, plant or viral were removed. Thus, yielding a contamination-free unpolished contig level assembly.

Haplotigs and either extremely high or extremely low coverage contigs were removed (using purge haplotigs (a-70))^36^. Remaining contigs were upgraded using FinisherSC^37^ and scaffolded using iterative rounds of SSPACE_longreads^38^ and gapFinisher^39^: Round 1 (error corrected reads, -k 1 -o 1000 -l 10 -g 500); round 2 (error corrected reads,-k 1 -o 1000 -l 5); and finally round 3 (raw reads, -k 1 -o 1000 -l 30). Scaffolds were then polished with three rounds of arrow (raw PacBio reads, smrttools-release_6.0.0.47835, in --diploid mode), and five rounds of Pilon (raw PacBio reads mapped with minimap2, 150 bp Illumina read pairs mapped with BWA-mem). This gave rise to assembly version 1.1.

During the initial phases of gene prediction on assembly version 1.1, it was noted when mapping RNAseq to the assembly that some SNPs in genes (often, but not always, A|G polymorphisms) were miscalled as adjacent indels (therefore altering the translation frame and negatively affecting gene calls). Mapped RNAseq was therefore used to create a variant call file (Pilon), which was parsed with a custom python script to identify all indels that were up to 4 bp apart (1451). All instances were inspected in a genome browser and corrected, giving rise to the final manually finished assembly version 1.2 (GenBank Accession JAHGVF000000000). Scripts, commands, and config files are available at: https://github.com/peterthorpe5/Heterodera_schachtii_genome_assembly and https://github.com/sebastianevda/H.schachtii_genome/assembly_and_manual_finishing_scripts).

### Gene prediction and annotation

Hard and soft genome masking was performed as described in^40, 41^. Briefly, Repeatmodeler (version DEV) was used to identify repetitive regions. The resulting identified repetitive elements were masked using RepeatMasker along with RepBaseRepeatMaskerEdition-20170127 models. To additionally identify transposons, TransposonPSI version 08222010^42^ and LTRharvest version 1.5.9^43^ from Genometools^44^ were used. All RNAseq reads were remapped to the soft masked genome, separated by strand, and used to provide stranded hints for Braker2 gene prediction (-f 2000, allowing Augustus to train itself for both exon and utr models). After considerable optimisation of extrinsic config files for exon and UTR prediction (available at https://github.com/sebastianevda/H.schachtii_genome/Gene_predictions), an extremely high-quality set of annotations resulted (termed annotation version 1). The version 1 predicted proteins were annotated with DIAMOND-BLASTp (version v0.9.24.125)^45^ searched against GenBank NR database. The resulting.xml file was imported into BLAST2GO version 5^46^. BLAST2GO was used to annotate the gene models using GO version May 9^th^ 2019 and Interproscan^47^. Gene duplication analyses were performed using the similarity searches from DIAMOND-BlastP (1 × 10^−10^) with MCSanX toolkit^17^. PFAM domains in protein-coding sequences were predicted using hmmsearch version 3.2.1^48^ and the Pfam-A.hmm definitions release 30 (http://ftp.ebi.ac.uk/pub/databases/Pfam/). Signal peptide and transmembrane prediction were performed using signalP 4.0^49^ and Phobius version 1.01^50^.

### Haplotype prediction

To represent the diploid genome and facilitate prediction of protein variants, haplotype phasing of large genome blocks was carried out using Whatshap v1.0 running in a Conda environment. The final *H. schachtii* reference genome v1.2 was used as input along with the 192X coverage PacBio read library and a variant call file generated with Pilon using the 144X coverage Illumina 150 bp paired-end reads. SNV sites with the same nucleotide in >90% of quality-filtered reads (Pilon QP score), sites marked by Pilon as Low Coverage, and sites for which Illumina read depth was <30 were removed from the input VCF file. A custom bash script (https://github.com/OlafKranse/ALT_caller_for_pilon) was used to reformat the filtered VCF file to include the second-most abundant nucleotide as the alternate allele (when the QP score for that nucleotide was >10% but <25%). Of the ~3.4 million variant calls that remained after quality filtering, there were 3709 nucleotide sites at which more than two variants were present, possibly representing third alleles that are present at lower allele frequencies (Supplementary Data 10). At an additional 3,106 sites, the second-most-abundant nucleotide call (QP score) was a tie between two different nucleotides (hence excluded from haplotype phasing but possibly representing second and third alleles; QP score typically ≤ 8%; Supplementary Data 11). A total of 601 sites identified by Pilon as carrying larger structural variants were not included in the reference genome or haplotypes and are reported in Supplementary Data 2. Minimap2 v2.22^51^ was used with the map-pb settings to align the PacBio reads to reference genome, followed by sorting and indexing using Samtools. The Whatshap phase command was then executed using default settings for a diploid genome, with the inclusion of indels^52^. A total of 3,338,269 of the 3,404,642 variant sites (Supplemental Files 1 DipphasedNewFinal.vcf) were thereby placed in 2,029 phased genomic blocks across the 395 contigs of the reference genome (average block size was 83,704 bp incorporating 1,645 variants; median block size was 27,417 bp incorporating 375 variants; longest block 2,310,618 bp). The two single haplotypes for each block were then placed (random choice) into one of two genome-wide files to generate the two additional *H. schachtii* genome resources Haplotype1 and Haplotype2 (Supplementary Data 12 and 13).

### Effector identification

Genes related to previously reported effectors in *G. rostochiensis* supported by gland cell expression were identified in the *H. schachtii* genome as previously^40^. In brief, an inclusive list of effectors was generated by sequence similarity alone (either BLAST (e-value <1 × 10^−5^) or using HMM searches (for glutathione synthetase-like effectors^6^ (PF03199.10 [http://pfam.xfam.org/family/PF03199] and PF03917.12 [http://pfam.xfam.org/family/PF03917]) and SPRY-domain (PF00622.23 [https://pfam.xfam.org/family/PF00622]) containing effectors^53^ (HMMsearch with gathering threshold cutoff)). Putative effector families were aligned and manually curated based on expert knowledge of effector characteristics (e.g. low scoring sequences without the appropriate effector domains for a particular effector family were removed). Gene models were manually inspected and re-annotated using Apollo where the gene prediction had failed to correctly capture the intron:exon structure of known effectors. Finally, putative effectors were cross-referenced with putative secreted proteins (i.e., encode a signal peptide but do not encode a transmembrane domain) to produce a high-confidence list of effectors (Supplementary Data 5).

### Horizontal gene transfer and phylogenetics

We used BLAST + to perform a similarity search of *H. schachtii* proteins against the nr database. Alien index (AI) was calculated with the Alienness web server^54^. We then used the AvP software (https://github.com/GDKO/AvP) to perform the HGT analyses. Proteins with AI above 10 were selected and grouped based on the percentage of shared BLAST hits (70%) using single linkage clustering. For each group we extracted the FASTA sequences of the BLAST hits from the NR database. Sequences for each group were aligned using MAFFT^55^ and FastTree^56^ was used for phylogenetic inference. In total 540 proteins (including alternative transcripts) were analysed, with 349 being confirmed as HGT candidates (222 of them had only hits from non-Metazoan taxa), 97 proteins had a non-conclusive topology, and in 94 proteins the hypothesis of being an HGT candidate was rejected. For the 127 proteins confirmed as HGT candidates that contained both Metazoan and non-Metazoan proteins, we performed a constrained phylogenetic inference enforcing the *H. schachtii* protein to form a monophyletic group with the other Metazoan proteins. We then performed tree topology tests with IQ-TREE 2^57^ and the associated approximately unbiased (AU) statistical test to check whether the likelihood of the constrained phylogenetic tree was significantly worse than that of the unconstrained phylogenetic tree. This analysis showed that in 30 cases both trees were equally likely, bringing down the total number of potential HGT candidates to 319 derived from 263 genes. For those sequences that could be tested, a p-value is included in Supplementary Data 6.

Nematode and other animal PANC sequences were collected from NCBI and wormbase parasite^58^ using the *H. schachtii* PANC as a query in BLAST. Probable contaminants were removed based on corresponding nucleotide identity >75% to non-Metazoans on NCBI and the absence of introns (the majority of non-nematode sequences were removed). Each remaining non-Metazoan PANC was used to identify the top 50 most similar sequences in NCBI using BLAST. A non-redundant list of subjects was aligned to all queries using MUSCLE, and refined^59^. The alignment was trimmed using Trimal (-gappyout^60^). Model selection (LG + F + I + G4) and phylogenetic inference (1000 bootstraps) was carried out using IQ-TREE 2^57^). The phylogenetic tree was midpoint re-rooted, and formatted using Figtree1.4.

### RNAseq read mapping, normalisation, differential gene expression analysis and clustering

RNAseq reads are available under Bioproject: PRJNA722882. All reads were analysed with FastQC v0.11.8^61^ and trimmed using BBduk v38.34 (https://sourceforge.net/projects/bbmap/). Only the reads with a minimum Phred Quality Score^62^ of 30 were kept. Based on the FastQC analysis, 10 nucleotides at the 5’ end of the reads were removed. The reads were also trimmed for the presence of adapters and only reads with a minimum length of 75 bp were used. The trimmed reads from each library were mapped onto the *A. thaliana* TAIR10 genome assembly combined with the *H. schachtii* 1.2 genome assembly using STAR^33^. The counting was performed only on the uniquely mapping reads using htseq-count part of HTseq v0.12.4^63^. The count tables were loaded in R v3.5.2^64^ and normalised with DESeq2 v1.22.2^65^. The clustering of each biological replicate was visualised by a Principal Component Analysis using the plotPCA function. Differentially expressed genes were identified using the DESeq2 v1.22.2 package following pairwise comparison between all samples (log2 fold change >0.5 and adjusted *p* value ≤0.01). The normalised expression of the differentially expressed genes was loaded then into MATLAB v9.6 R2019a and clustered using the tcap2 plugin^66^. The clustering tables were then loaded and analysed in R using the tidyverse package v1.2.1^67^. Differential expression clusters with different magnitudes but similar profiles were manually grouped into 30 and 29 biologically relevant expression superclusters for the host and parasite respectively. Visualisations were obtained using the R package ggplot2 v3.1.0. All normalised expression data are available in Supplementary Data 4 and 7.

### OrthoMCl analyses

Orthogroup analyses were precomputed and already available for plants and nematodes. For plants, we focused on a subset of eight plant species (*Amborella trichopoda* (outgroup), the monocots *Hordeum vulgare* and *Zea mays*, the solanaceous *Solanum lycopersicum* and *S. tuberosum*, the fabaceous *Glycine max*, and the brassicaceous *Brassica* rapa (ssp. *rapa*) and *A. thaliana* - GreenPhyl v5 - MCL matrix for level 4 clusters^68^). Counts per species per orthogroup were generated using a custom python script (https://github.com/sebastianevda/H.schachtii_genome/tree/main/MCL_parsing). Orthogroups, or subsets of orthogroups that contain genes of interest (e.g., genes in a particular expression cluster), were visualised using UpSetR R package. For nematodes, we focused on a subset of eight nematodes species (the free living nematode *Caenorhabditis elegans* (outgroup), the pine wilt nematode *Bursaphelenchus xylophilus*, the root-knot nematodes *Meloidogyne graminicola* and *M. hapla*, the potato cyst nematodes *Globodera pallida* and *G. rostochiensis*, and the *Heterodera* species, *H. glycines* and *H. schachtii*^18^).

### KEGG mapping

The KEGG Automatic Annotation Server Ver. 2.1 (https://www.genome.jp/kaas-bin/kaas_main) was used to annotate metabolic processes in the host and the parasite using the bi-directional best hit strategy (using the default dataset and parameters with the following exception: minimum bit score increased to 100, Supplementary Data 9). The KEGG reconstruct server was used to populate existing pathways with metabolic process information.

### Nematode infection assays

Cysts of *H. schachtii* were harvested from monoculture of mustard (*Sinapsis alba* ‘Albatros’) plants growing on modified Knop nutrient medium (2% w/v). The hatching of the nematodes was stimulated by adding 3 mM ZnCl2. Arabidopsis plants were grown in Petri dishes containing agar supplemented with Knop nutrient medium (2% w/v) under sterile conditions. Two plants were grown in one Petri dish. 60−70 J2s were inoculated to the surface of agar medium containing 12-days-old Arabidopsis plants. The numbers of nematodes per plant were counted using a stereomicroscope (Leica Microsystems) at 14 dpi. The female nematodes and female‐associated syncytia were outlined, and the area was calculated using an M165C stereomicroscope equipped with LAS v. 4.3 image analysis software (Leica Microsystems) at 14 dpi. Cyst sizes were outlined, and the area was calculated using an M165C stereomicroscope equipped with LAS v. 4.3 image analysis software (Leica Microsystems) at 42 dpi. Cysts were randomly selected and crushed and were then transferred into a counting dish and the number of eggs/cysts were counted using a stereomicroscope (Leica Microsystems). All infection assays were repeated a minimum of three times.

### In situ hybridisation

Using *H. schachtii* cDNA as template, digoxigenin (DIG)-labelled probes complementary to a target sequence in *Hs-panc1* were amplified in asymmetric PCR. The PCR was performed with single sense (negative control) or antisense primers in presence of DIG-labelled deoxynucleotide triphosphates (dNTPs) (Roche). Freshly-hatched J2s were fixed and hybridised with the probes following the protocol of^69^. The hybridised nematodes then were examined using Leica DMI2000 compound microscope. Primers are listed in Supplementary Table 8.

### Light and electron microscopy

*A. thaliana* line Col-0 and *atpanb1-1* mutant were grown aseptically in Petri dishes on KNOP medium (Sijmons et al. 1991). Two-weeks old plants were inoculated with about 100 freshly-hatched sterile J2s of beet cyst nematode (*H. schachtii*) as described above. Samples consisting of root pieces containing syncytia and attached juveniles were collected 5 and 10 dpi. After dissection, they were immediately transferred into a fixative consisting of 2% (w/v) paraformaldehyde (Sigma-Aldrich, St. Louis, MI, USA) and 2% (v/v) glutaraldehyde (Sigma-Aldrich) dissolved in 0.05 M sodium cacodylate (Sigma-Aldrich) buffer (pH = 7.2). After 2 hours, the fixative was replaced with excess buffer 4 times, each with 2 hour incubation. Samples were post-fixed in 2% (w/v) aqueous solution of osmium tetroxide (Sigma-Aldrich) for 2 hours and washed in the cacodylic buffer. They were dehydrated in ascending series of ethanol solutions (up to 99.8%), which was replaced with propylene oxide for 1 hour. They were then incubated in ascending series of EPON epoxy resin mixtures dissolved with propylene oxide. After incubation in pure EPON resin the samples were singly transferred into flat embedding moulds and the resin was cured at 65 °C for 16 h. For light microscopy examinations, 3 µm thick sections were cut on a Leica RM2165 microtome (Leica, Wetzlar, Germany) and stained with 0.1% (w/v) aqueous solution of Toluidine Blue (pH = 6.9, Sigma-Aldrich). Sections were examined under an Olympus AX70 ‘Provis’ microscope (Olympus, Tokyo, Japan) operating in a bright field mode and equipped with an Olympus UC90 (Olympus) digital camera. Sections (ca. 90 nm thick) for transmission electron microscopy examinations were cut with a Leica Ultracut UCT (Leica) ultramicrotome. They were collected on single-slot formvar-coated copper grids and contrasted with a saturated ethanolic solution of uranyl acetate (Sigma-Aldrich) for 15 min and an aqueous solution of lead citrate (Sigma-Aldrich) for 10 min. Sections were examined under an FEI 256D ‘Morgagni’ (Thermo Fisher Scientific, Watham, MA, USA) transmission electron microscope operating at 80 kV and equipped with an Olympus-SIS ‘Morada’ (Olympus) digital camera. Acquired digital images were cropped, resized and adjusted for similar contrast and brightness using Adobe software package (Adobe Inc., San Jose, CA, USA).

### Generation of overexpression and complementation lines

Full-length coding sequence of *AtPANB1* (AT2G46110) was amplified from cDNA synthesised from RNA isolated from 12-days-old Arabidopsis plants. The amplified sequence was cloned into Gateway cloning vector pDONR207 (Invitrogen, USA) using the Gateway® BP Clonase II Enzyme mix (Invitrogen). The cloned fragments were verified through sequencing and transferred via Gateway recombination into the pMDC32 vector under the control of *35* *S* promoter. The verified constructs were introduced into *Agrobacterium tumefaciens* strain GV3101, which was used for the transformation of 4- to 6-week-old Col-0 or *atpanb1-1* mutant plants by the floral dip method (Clough and Bent 1998). After drying of plants, seeds (T0) were harvested and sterilised before being sown on Knop medium supplemented with 25 µg ml^−1^ hygromycin. Transformants were selected to produce homozygous plants. Two to three independent homozygous lines with the highest upregulation were selected for further studies.

### Gene silencing

We identified two 21-nt long target sequences in *Hs-panc2* mRNA that begin with an AA dinucleotide. Sense and antisense oligonucleotides for the 21-nt target sequences were obtained in which the Us were replaced with Ts. The 8-nt long sequence (5’-CCTGTCTC-3’) complementary to the T7 promoter primer was added to the 3’ ends of both the sense and antisense oligonucleotides. Afterwards, dsRNA was synthesised from the sense and antisense oligonucleotides using Silencer® siRNA Construction Kit (cat. no. AM1620) according to the manufacturer’s instructions. The siRNA targeting *eGFP* was used as a control. Approximately 2000 nematodes were incubated in a dsRNA solution containing siRNA, 3 mM spermidine, and 50 mM octopamine. After 24 h incubation, nematodes were surface-sterilised in 0.05% (w/v) HgCl_2_, and infected on 12-day-old Col-0 Arabidopsis plants. Each plant was inoculated with 60–70 J2s. Approximately, 300–500 J2s were used for RT-qPCR as described below.

### Real-time quantitative PCR

RNA was extracted using a Nucleospin RNA XS (Macherey-Nagel, Germany) kit according to the manufacturer’s protocol. RNA was treated with DNase1 using a DNA-free^TM^ DNA Removal Kit (Ambion) to remove contaminating DNA. cDNA was synthesised using a High Capacity cDNA Reverse Transcription Kit (Life-Technologies, cat. no. 4368814) according to the manufacturer’s instructions. Transcript abundance was measured using the StepOnePlus Real-Time PCR System (Applied Biosynthesis, USA). Each sample contained 10 μl of Fast SYBR Green qPCR Master Mix with uracil-DNA, glycosylase, and 6-carboxy-x-rhodamine (Invitrogen), 2 mM MgCl_2_, 0.5 μl each of forward and reverse primers (10 μM), 2 μl of complementary DNA (cDNA), and water in 20 μl total reaction volume. Data shown are an average of three independent biological replicates. Each biological replicate consisted of 2–3 technical replicates. *18* *S* was used as an internal control except for nematode samples. For nematode samples, actin was used as an internal control. cDNA was diluted 1:100 for *18* *S* amplification. Relative expression was calculated by Pfaffl’s method^70^ where the expression of the target gene was normalised to internal control to calculate the fold change. All primers are listed in Supplementary Table 8 and uncropped scans of gels are available in the supplementary information file (Supplementary Fig. 8).

### Genotyping and expression analysis of knockout mutants

T-DNA insertion mutants were ordered from the Nottingham Stock Centre (*atpanb1-1*, SALK_18243C; *atpanc*, Salk_101909C). The homozygous lines were checked for lack of expression through RT-PCR using primers listed in Supplementary Table 8. Loss-of-function for *atpanc* (SALK_101909C) has been previously published^26^; homozygous plants show an embryo-lethal phenotype, which can be rescued by exogenous pantothenate. *atpanc* showed inconsistent phenotype in our growth medium both in terms of root growth and infection.

### Oxidative burst assay

The measurement of ROS production was carried out via a luminol-based method according to Mendy et al.^71^. Leaf disks measuring 0.5 cm in diameter were cut from 12-days-old Arabidopsis plants grown on the soil under greenhouse conditions. Leaf disks were incubated in water for 12 h. After incubation, each leaf disk was transferred to a well in a 96-well plate containing 15 μl of 20 μg ml^−1^ horseradish peroxidase and 35 μl of 10 mM 8-amino-5 chloro-2,3-dihydro-7phenyl(3,4-d) pyridazine sodium salt (L-012, Wako Chemicals). Next, 50 μl of either flg22 or water was added to individual wells. Light emission was measured as relative light units in a 96-well luminometer (Mithras LB 940; Berthold Technologies) over 60 min and analysed using instrument software and Microsoft Office Excel. The experiments were performed in nine biological replicates.

### Pathogenicity assays

The plant susceptibility to *Botrytis cinerea* was evaluated via a modified protocol according to Lozano-Torres et al.^72^. Briefly, 5 μl drops of conidial suspensions (5 × 10^5^) were placed on the leaves of 4-week-old Arabidopsis plants were grown on the soil under the greenhouse conditions. Five microlitre drops of conidial suspensions (5 × 10^5^) were placed on the leaves of 4-week-old plants. After inoculation, plants were placed in the dark at 20 °C and 100% relative humidity for 3 days. Next, leaves containing necrotic lesions were cut and the necrotic area was measured. *Serendipita indica* assays were performed according to Daneshkhah et al.^73^. Five millimetre S*. indica* mycelium plugs, grown at 28 °C on CM medium, were inoculated to 7 days-old Arabidopsis seedlings grown on MS medium (Duchefa Biochemie, The Netherlands). Fresh roots and shoot weights were measured 7 days after inoculation.

### Bacterial complementation assay

Functional complementation analysis was performed in *E. coli* ∆panC mutants AT1371 (auxotroph for vitamin B5). Full-length ORFs of *AtPANC*, *Ec-panc*, and *Hs-panc* were synthesised by Genewiz LLC and cloned into pUC18 vectors. The amplified fragments were cloned in a frame with the *lacZ α* gene (encoding α peptide of β-galactosidase) under the control of an inducible *lacZ* promoter. All the constructs were transferred into the competent cells of AT1371 by heat shock method. Transformants carrying pUC18 empty vector were used as a negative control. The transformants were grown overnight on LB agar medium containing Ampicillin (100 µg ml^−1^). Positive clones were selected by colony PCR. A growth curve assay was performed by growing transformants overnight in LB medium. A loop of cells was added to M63 minimal media broth devoid of D-pantothenic acid but supplemented with Ampicillin (100 µg ml^−1^) and IPTG (1 mM). Cultures were grown at 37 °C at 120 rpm for 24–72 h. The growth of the bacteria was assayed in terms of optical density (OD600 nm) of the cultures.

### Reporting summary

Further information on research design is available in the Nature Research Reporting Summary linked to this article.

## Supplementary information

Supplementary information file

Reporting Summary

Description of Additional Supplementary Files

Supplementary Dataset 1

Supplementary Dataset 2

Supplementary Dataset 3

Supplementary Dataset 4

Supplementary Dataset 5

Supplementary Dataset 6

Supplementary Dataset 7

Supplementary Dataset 8

Supplementary Dataset 9

Supplementary Dataset 10

Supplementary Dataset 11

Supplementary Dataset 12

Supplementary Dataset 13

## Data Availability

The Whole-Genome Shotgun data generated in this study have been deposited in the DDBJ/ENA/GenBank database under accession code JAHGVF010000000. The Genomic and RNAseq data generated in this study have been deposited in the DDBJ/ENA/GenBank database under accession code Bioproject: PRJNA722882. The Host expression data generated in this study are available in the Arabidopsis ePlant browser (https://bar.utoronto.ca/eplant/). The processed gene expression data generated in this study are available in the Supplementary Data associated with this manuscript. The Gene calls, proteins, etc. are available on wormbaseparasite (https://parasite.wormbase.org/index.html). Source data are provided with this paper.

## Source data

Source Data File

## Code availability

Code and scripts used in the manuscript are available here: https://github.com/sebastianevda/H.schachtii_genome (ref.^74^), and https://github.com/OlafKranse/ALT_caller_for_pilon (ref.^75^), and https://github.com/peterthorpe5/Heterodera_schachtii_genome_assembly (ref.^76^).

## References

[1] Savary S (2019). The global burden of pathogens and pests on major food crops. Nat. Ecol. Evol..

[2] Abd-Elgawad, M. M. M., et al. Impact of phytonematodes on agriculture economy. in *Biocontrol agents of phytonematodes* 3–49 (CAB International, 2015).

[3] Nicol, J. M. et al. Current Nematode Threats to World Agriculture. in *Genomics and Molecular Genetics of Plant-Nematode Interactions* (eds. Jones, J., Gheysen, G. & Fenoll, C.) 21–43 (2011).

[4] Baunacke, W. *Untersuchungen zur Biologie und Bekämpfung der Rübennematoden Heterodera schachtii Schmidt*. (P. Parey, 1922).

[5] Wyss U (1992). Observations on the feeding behaviour of Heterodera schachtii throughout development including events during moulting. Fundam. Appl. Nematol..

[6] Lilley CJ (2018). Effector gene birth in plant parasitic nematodes: neofunctionalization of a housekeeping glutathione synthetase gene. PLoS Genet..

[7] Eves-van den Akker S, Lilley CJ, Jones JT, Urwin PE (2014). Identification and characterisation of a hyper-variable apoplastic effector gene family of the potato cyst nematodes. PLoS Pathog..

[8] Szakasits D (2009). The transcriptome of syncytia induced by the cyst nematode Heterodera schachtii in Arabidopsis roots. Plant J..

[9] Golinowski W, Grundler FMW, Sobczak M (1996). Changes in the structure ofArabidopsis thaliana during female development of the plant-parasitic nematodeHeterodera schachtii. Protoplasma.

[10] Sobczak M, Golinowski W, Grundler FMW (1997). Changes in the structure of Arabidopsis thaliana roots induced during development of males of the plant parasitic nematode Heterodera schachtii. Eur. J. Plant Pathol..

[11] Grundler FMW, Sobczak M, Golinowski W (1998). Formation of wall openings in root cells of Arabidopsis thaliana following infection by the plant-parasitic nematode Heterodera schachtii. Eur. J. Plant Pathol..

[12] Raski DJ (1950). The life history and morphology of the sugar-beet nemarode, Heteroderaschacktii Schmidt. Phytopathology.

[13] Grundler F, Betka M, Wyss U (1991). Influence of changes in the nurse cell system (syncytium) on sex determination and development of the cyst nematode Heterodera schachtii: total amounts of proteins and amino acids. Phytopathology.

[14] Anjam MS (2020). Host factors influence the sex of nematodes parasitizing roots of Arabidopsis thaliana. Plant Cell Environ..

[15] Yan G, Baidoo R (2018). Current research status of heterodera glycines resistance and its implication on soybean breeding. Proc. Est. Acad. Sci. Eng..

[16] Kumar, A. et al. A rhomboid-like protease gene from an interspecies translocation confers resistance to cyst nematodes. *New Phytol*. **231**, 801–813 (2021). 10.1111/nph.17394 33866563

[17] Wang Y (2012). MCScanX: a toolkit for detection and evolutionary analysis of gene synteny and collinearity. Nucleic Acids Res..

[18] Grynberg, P. et al. Comparative genomics reveals novel target genes towards specific control of plant-parasitic nematodes. *Genes* **11**, 1347 (2020). 10.3390/genes11111347 PMC7696266 33202889

[19] Cotton JA (2014). The genome and life-stage specific transcriptomes of Globodera pallida elucidate key aspects of plant parasitism by a cyst nematode. Genome Biol..

[20] Craig JP, Bekal S, Niblack T, Domier L, Lambert KN (2009). Evidence for horizontally transferred genes involved in the biosynthesis of vitamin B(1), B(5), and B(7) in Heterodera glycines. J. Nematol..

[21] Wybouw N, Van Leeuwen T, Dermauw W (2018). A massive incorporation of microbial genes into the genome of Tetranychus urticae, a polyphagous arthropod herbivore. Insect Mol. Biol..

[22] Holbein J (2019). Root endodermal barrier system contributes to defence against plant-parasitic cyst and root-knot nematodes. Plant J..

[23] Jones JT (2013). Top 10 plant-parasitic nematodes in molecular plant pathology. Mol. Plant Pathol..

[24] Genschel U, Powell CA, Abell C, Smith AG (1999). The final step of pantothenate biosynthesis in higher plants: cloning and characterization of pantothenate synthetase from Lotus japonicus and Oryza sativum (rice). Biochem. J..

[25] Ronconi, S. The pantothenic acid and coenzyme A pathway in plants and archaea. (Technische Universität München, 2006).

[26] Jonczyk R, Genschel U (2006). Molecular adaptation and allostery in plant pantothenate synthetases. J. Biol. Chem..

[27] Miyatake K, Nakano Y, Kitaoka S (1979). Pantothenate synthetase from Escherichia coli [D-pantoate: beta-alanine ligase (AMP-forming), EC 6.3.2.1]. Methods Enzymol..

[28] Zheng R, Blanchard JS (2001). Steady-state and pre-steady-state kinetic analysis of Mycobacterium tuberculosis pantothenate synthetase. Biochemistry.

[29] Koren S (2017). Canu: scalable and accurate long-read assembly via adaptive k-mer weighting and repeat separation. Genome Res..

[30] Ruan J, Li H (2020). Fast and accurate long-read assembly with wtdbg2. Nat. Methods.

[31] Simão FA, Waterhouse RM, Ioannidis P, Kriventseva EV, Zdobnov EM (2015). BUSCO: assessing genome assembly and annotation completeness with single-copy orthologs. Bioinformatics.

[32] Stanke M (2006). AUGUSTUS: ab initio prediction of alternative transcripts. Nucleic Acids Res..

[33] Dobin A (2013). STAR: ultrafast universal RNA-seq aligner. Bioinformatics.

[34] Parra G, Bradnam K, Korf I (2007). CEGMA: a pipeline to accurately annotate core genes in eukaryotic genomes. Bioinformatics.

[35] Laetsch DR, Blaxter ML (2017). BlobTools: Interrogation of genome assemblies. F1000Res..

[36] Roach MJ, Schmidt SA, Borneman AR (2018). Purge Haplotigs: allelic contig reassignment for third-gen diploid genome assemblies. BMC Bioinform..

[37] Lam K-K, LaButti K, Khalak A, Tse D (2015). FinisherSC: a repeat-aware tool for upgrading de novo assembly using long reads. Bioinformatics.

[38] Boetzer M, Pirovano W (2014). SSPACE-LongRead: scaffolding bacterial draft genomes using long read sequence information. BMC Bioinform..

[39] Kammonen JI (2019). gapFinisher: a reliable gap filling pipeline for SSPACE-LongRead scaffolder output. PLoS One.

[40] Eves-van den Akker S (2016). The genome of the yellow potato cyst nematode, Globodera rostochiensis, reveals insights into the basis of parasitism and virulence. Genome Biol..

[41] Thorpe P, Escudero-Martinez CM, Cock PJA, Eves-van den Akker S, Bos JIB (2018). Shared transcriptional control and disparate gain and loss of aphid parasitism genes. Genome Biol. Evol..

[42] Haas, B. TransposonPSI: an application of PSI-Blast to mine (retro-) transposon ORF homologies. Broad Institute, Cambridge, MA, USA (2007).

[43] Ellinghaus D, Kurtz S, Willhoeft U (2008). LTRharvest, an efficient and flexible software for de novo detection of LTR retrotransposons. BMC Bioinform..

[44] Gremme G, Steinbiss S, Kurtz S (2013). GenomeTools: a comprehensive software library for efficient processing of structured genome annotations. IEEE/ACM Trans. Comput. Biol. Bioinform..

[45] Buchfink B, Xie C, Huson DH (2015). Fast and sensitive protein alignment using DIAMOND. Nat. Methods.

[46] Conesa A (2005). Blast2GO: a universal tool for annotation, visualization and analysis in functional genomics research. Bioinformatics.

[47] Quevillon E (2005). InterProScan: protein domains identifier. Nucleic Acids Res..

[48] Eddy SR (2011). Accelerated profile HMM searches. PLoS Comput. Biol..

[49] Petersen TN, Brunak S, von Heijne G, Nielsen H (2011). SignalP 4.0: discriminating signal peptides from transmembrane regions. Nat. Methods.

[50] Käll L, Krogh A, Sonnhammer ELL (2004). A combined transmembrane topology and signal peptide prediction method. J. Mol. Biol..

[51] Li H (2018). Minimap2: pairwise alignment for nucleotide sequences. Bioinformatics.

[52] Patterson M (2015). WhatsHap: weighted haplotype assembly for future-generation sequencing reads. J. Comput. Biol..

[53] Rehman S (2009). A secreted SPRY domain-containing protein (SPRYSEC) from the plant-parasitic nematode Globodera rostochiensis interacts with a CC-NB-LRR protein from a susceptible tomato. Mol. Plant. Microbe Interact..

[54] Rancurel C, Legrand L, Danchin E (2017). Alienness: rapid detection of candidate horizontal gene transfers across the tree of life. Genes.

[55] Katoh K, Standley DM (2013). MAFFT multiple sequence alignment software version 7: improvements in performance and usability. Mol. Biol. Evol..

[56] Price MN, Dehal PS, Arkin AP (2010). FastTree 2—approximately maximum-likelihood trees for large alignments. PLoS One.

[57] Minh BQ (2020). IQ-TREE 2: new models and efficient methods for phylogenetic inference in the genomic era. Mol. Biol. Evol..

[58] Howe KL, Bolt BJ, Shafie M, Kersey P, Berriman M (2017). WormBase ParaSite − a comprehensive resource for helminth genomics. Mol. Biochem. Parasitol..

[59] Edgar RC (2004). MUSCLE: multiple sequence alignment with high accuracy and high throughput. Nucleic Acids Res..

[60] Capella-Gutiérrez S, Silla-Martínez JM, Gabaldón T (2009). trimAl: a tool for automated alignment trimming in large-scale phylogenetic analyses. Bioinformatics.

[61] Andrews, S. et al. FastQC: a quality control tool for high throughput sequence data. (2010).

[62] Ewing B, Hillier L, Wendl MC, Green P (1998). Base-calling of automated sequencer traces using phred. I. Accuracy assessment. Genome Res..

[63] Anders S, Pyl PT, Huber W (2014). HTSeq—a Python framework to work with high-throughput sequencing data. Bioinformatics.

[64] Computing, R. et al. R: a language and environment for statistical computing. *R Core Team* (2013).

[65] Love MI, Huber W, Anders S (2014). Moderated estimation of fold change and dispersion for RNA-seq data with DESeq2. Genome Biol..

[66] Kiddle SJ (2010). Temporal clustering by affinity propagation reveals transcriptional modules in Arabidopsis thaliana. Bioinformatics.

[67] Wickham H (2019). Welcome to the tidyverse. J. Open Source Softw..

[68] Guignon V (2021). GreenPhylDB v5: a comparative pangenomic database for plant genomes. Nucleic Acids Res..

[69] de Boer JM, Yan Y, Smant G, Davis EL, Baum TJ (1998). In situ hybridization to messenger RNA in Heterodera glycines. J. Nematol..

[70] Pfaffl MW (2001). A new mathematical model for relative quantification in real-time RT-PCR. Nucleic Acids Res..

[71] Mendy B (2017). Arabidopsis leucine-rich repeat receptor-like kinase NILR1 is required for induction of innate immunity to parasitic nematodes. PLoS Pathog..

[72] Lozano-Torres JL (2014). Apoplastic venom allergen-like proteins of cyst nematodes modulate the activation of basal plant innate immunity by cell surface receptors. PLoS Pathog..

[73] Daneshkhah R (2013). Piriformospora indica antagonizes cyst nematode infection and development in Arabidopsis roots. J. Exp. Bot..

[74] Eves-van den Akker, S. The genome and lifestage-specific transcriptomes of a plant-parasitic nematode and its host reveal susceptibility genes involved in trans-kingdom synthesis of vitamin B5. *Github*. 10.5281/zenodo.7074704. (2022). 10.1038/s41467-022-33769-w PMC9582021 36261416

[75] Kranse, O. P. The genome and lifestage-specific transcriptomes of a plant-parasitic nematode and its host reveal susceptibility genes involved in trans-kingdom synthesis of vitamin B5. *Github*. 10.5281/zenodo.7074747. (2022) 10.1038/s41467-022-33769-w PMC9582021 36261416

[76] Thorpe, P. The genome and lifestage-specific transcriptomes of a plant-parasitic nematode and its host reveal susceptibility genes involved in trans-kingdom synthesis of vitamin B5. *Github*. 10.5281/zenodo.7085297. (2022). 10.1038/s41467-022-33769-w PMC9582021 36261416

