# Supplementary material for: The genome and lifestage-specific transcriptomes of a plant-parasitic nematode and its host reveal susceptibility genes involved in trans-kingdom synthesis of vitamin B5
Source: Nat Commun. 2022 Oct 19;13:6190. doi: 10.1038/s41467-022-33769-w (PMC9582021; doi:10.1038/s41467-022-33769-w)
Supplement: Supplementary file 1 — Supplementary information file [file 41467_2022_33769_MOESM1_ESM.pdf]

Supplementary Figures

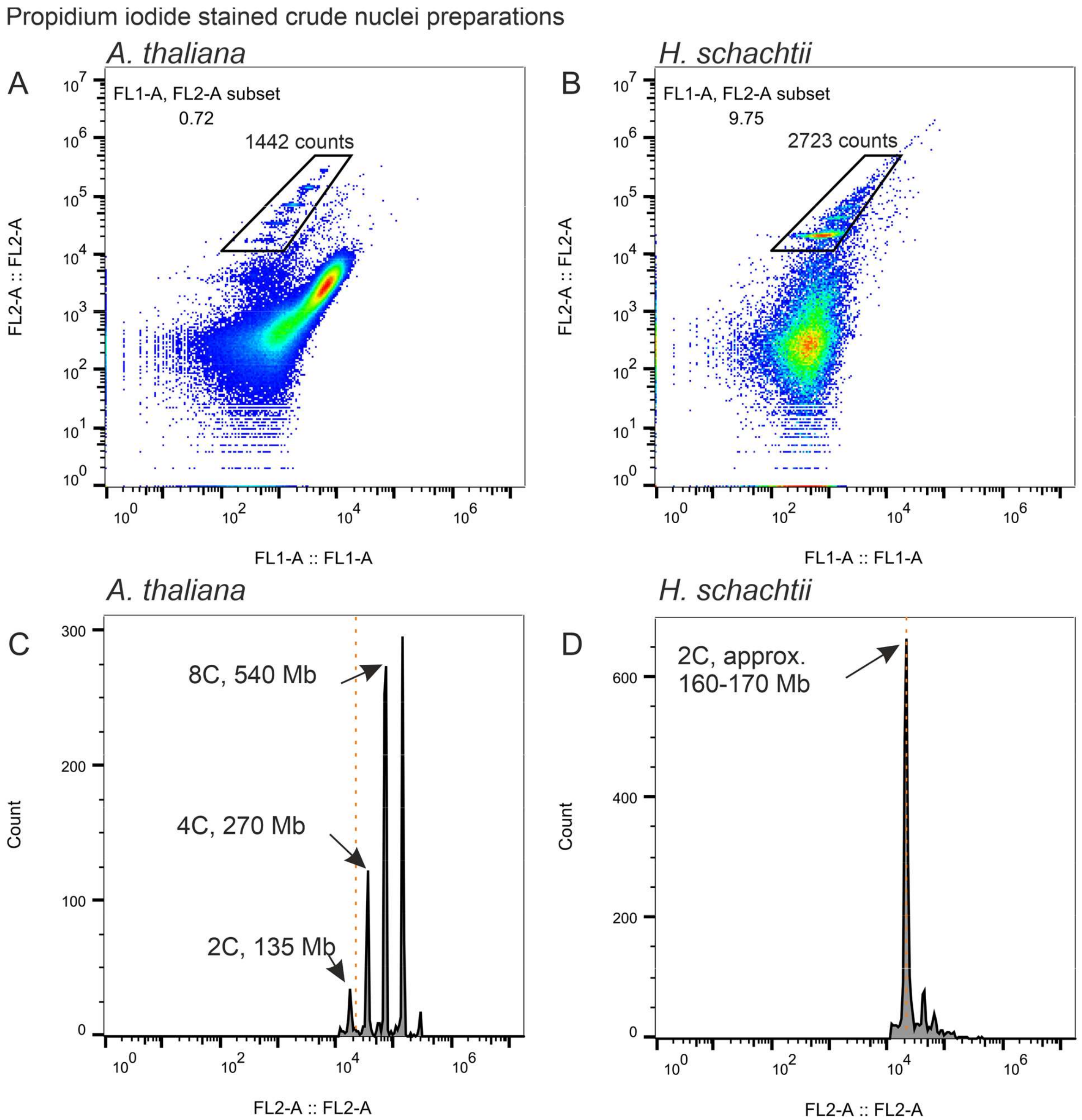

Supplemental Figure 1. Flow cytometric estimation of genome size in *H. schachtii*. A and B) Pseudo colour plots showing gating strategy for *A. thaliana* (Col-0 population) characteristic leaf endoreduplication (left), mirrored to *H. schachtii* (right) respectivley. C and D) Histogram counts of nuclei within the gate for *A. thaliana* and *H. schachtii* respectively. E) Overlay of smoothed histograms for *A. thaliana* (green, bottom) and *H. schachtii* (red, top). Orange dotted overlay indicates the same point on each graph to facilitate comparison. Numbers in Mb represent genome length of collapsed haplotypes of each species. A corresponds to C, B corresponds to D.

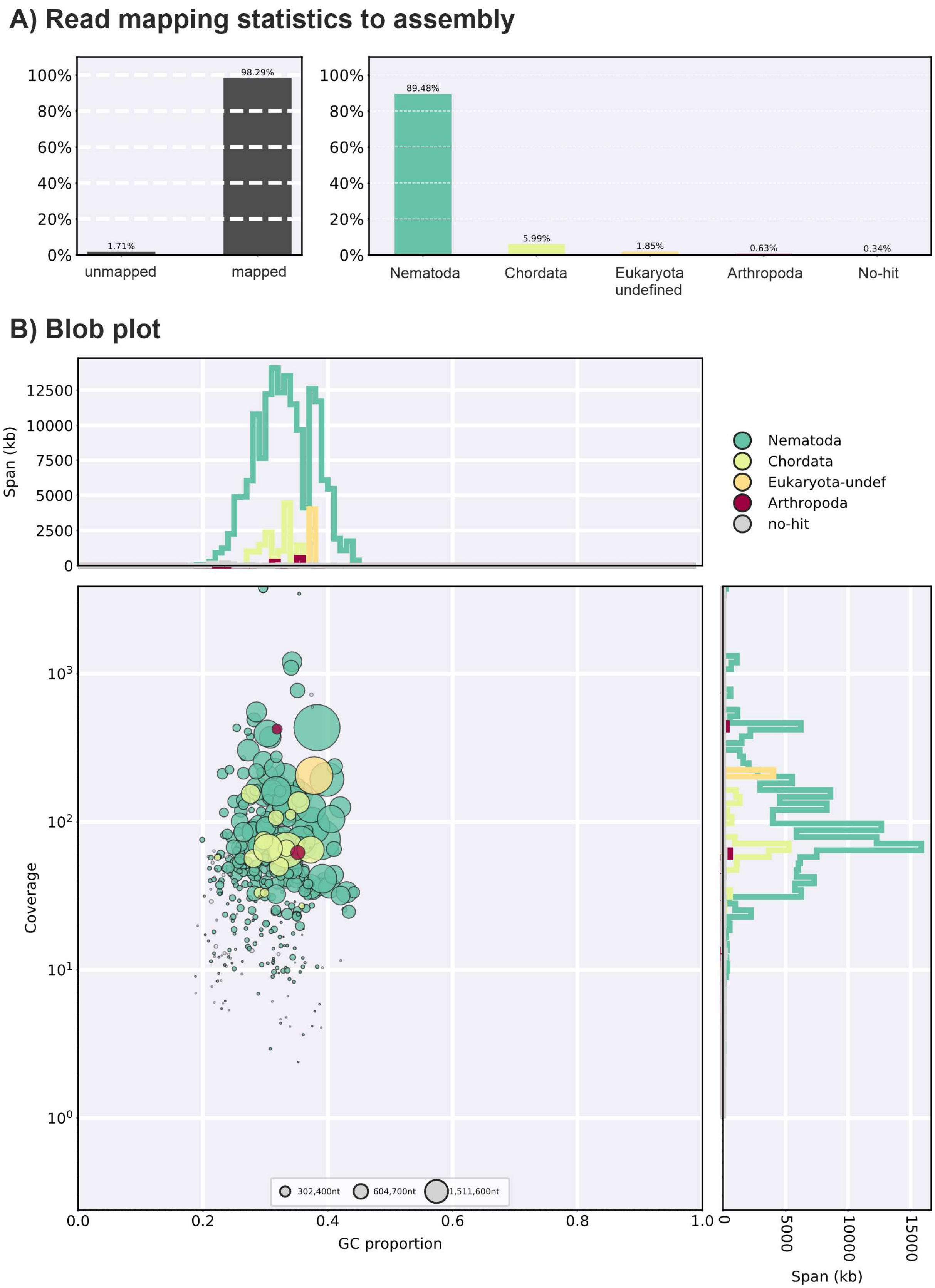

Supplemental Figure 2. Blob plot contamination assessment of final assembly. A) Left, read mapping proportions to the final assembly of the *H. schachtii* genome. Right, distribution of mapped reads by taxonomic group. **B**) Two-dimensional scatter plots, decorated with coverage and GC histograms. Circles are coloured by taxonomic group.

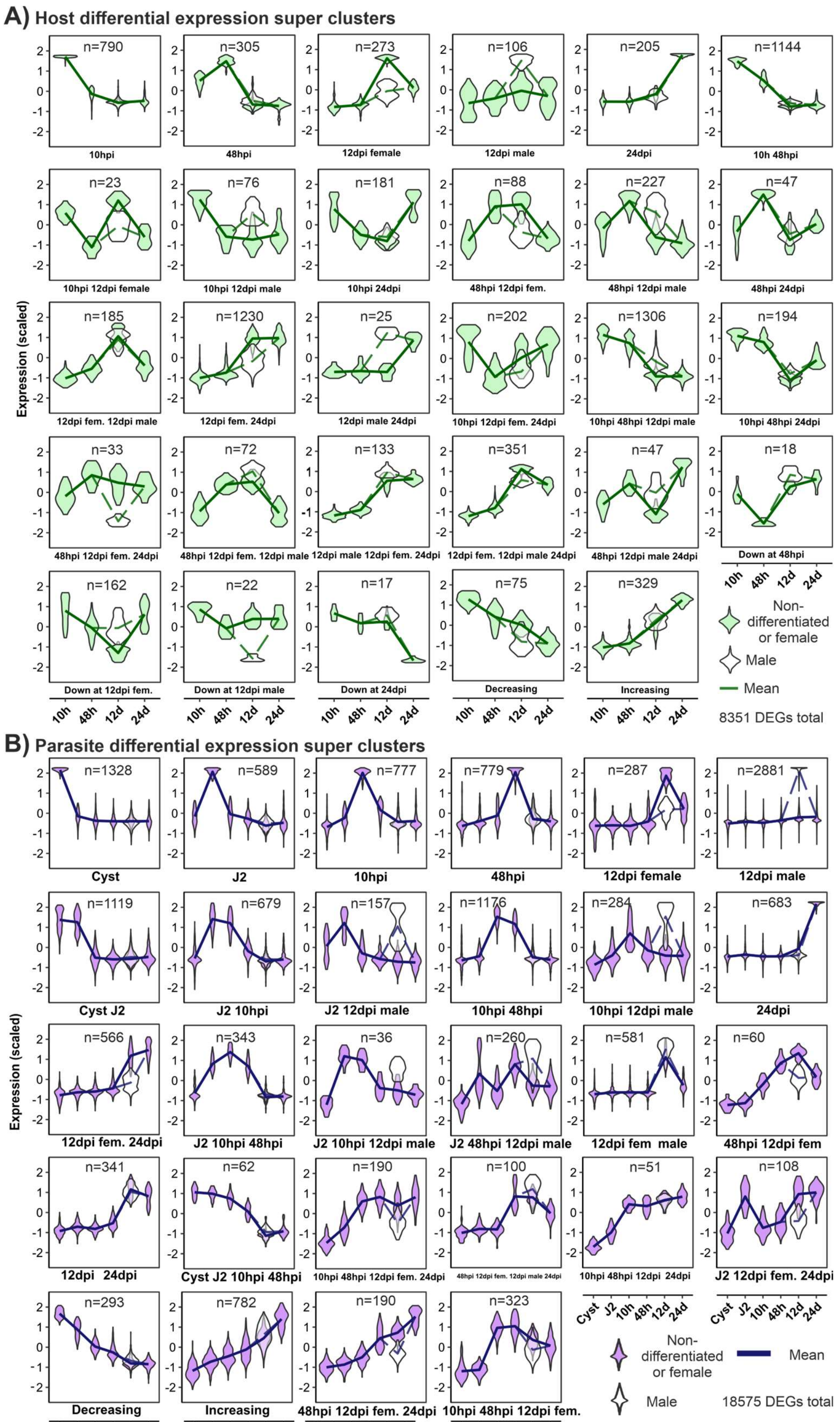

Supplemental Figure 3. All differentially expressed super clusters. Expression super clusters for host (**A**) and parasite (**B**). Each violin subplot shows centered expression for genes in the cluster. The mean of each life stage is represented with a line. Open violins show gene expression for the 12 dpi male (or syncytial gene expression associated with it). Closed violins show gene expression of all other life stages (or the host gene expression associated with them). Numbers and proportions of genes in each cluster are available in Supplemental Tables 4 and 5.

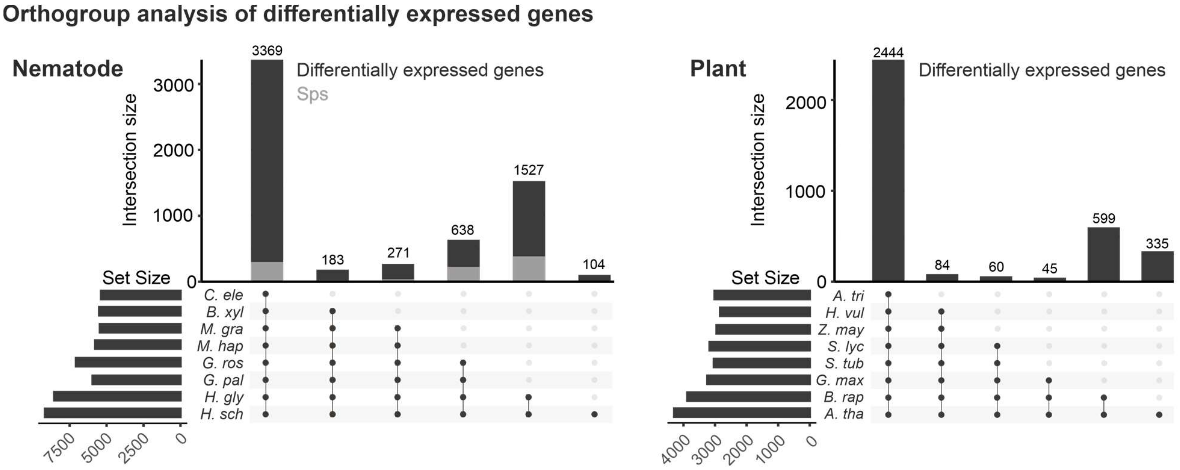

Supplemental Figure 4. Orthogroup analysis of host and parasite. Upset graphs of orthologous gene clusters between eight nematode (left - *Caenorhabditis elegans, Bursaphelenchus xylophilus*, *Meloidogyne graminicola, M. hapla*, *Globodera pallida, G. rostochiensis*, *Heterodera glycines,* and *H. schachtii*) and eight plant species (right - *Amborella trichopoda, Hordeum vulgare*, *Zea mays*, *Solanum lycopersicum, S. tuberosum*, *Glycine max*, *Brassica rapa* (ssp. *rapa*) and *A. thaliana*). The intersections for 6 categories are shown. For example, there are 104 orthogroups that exclusively contain sequences from *H. schachtii*. Orthogroups that contain putatively secreted proteins (Sps) are indicated in grey.

Contrasting histories of host and parasite genes up-regulated during infection

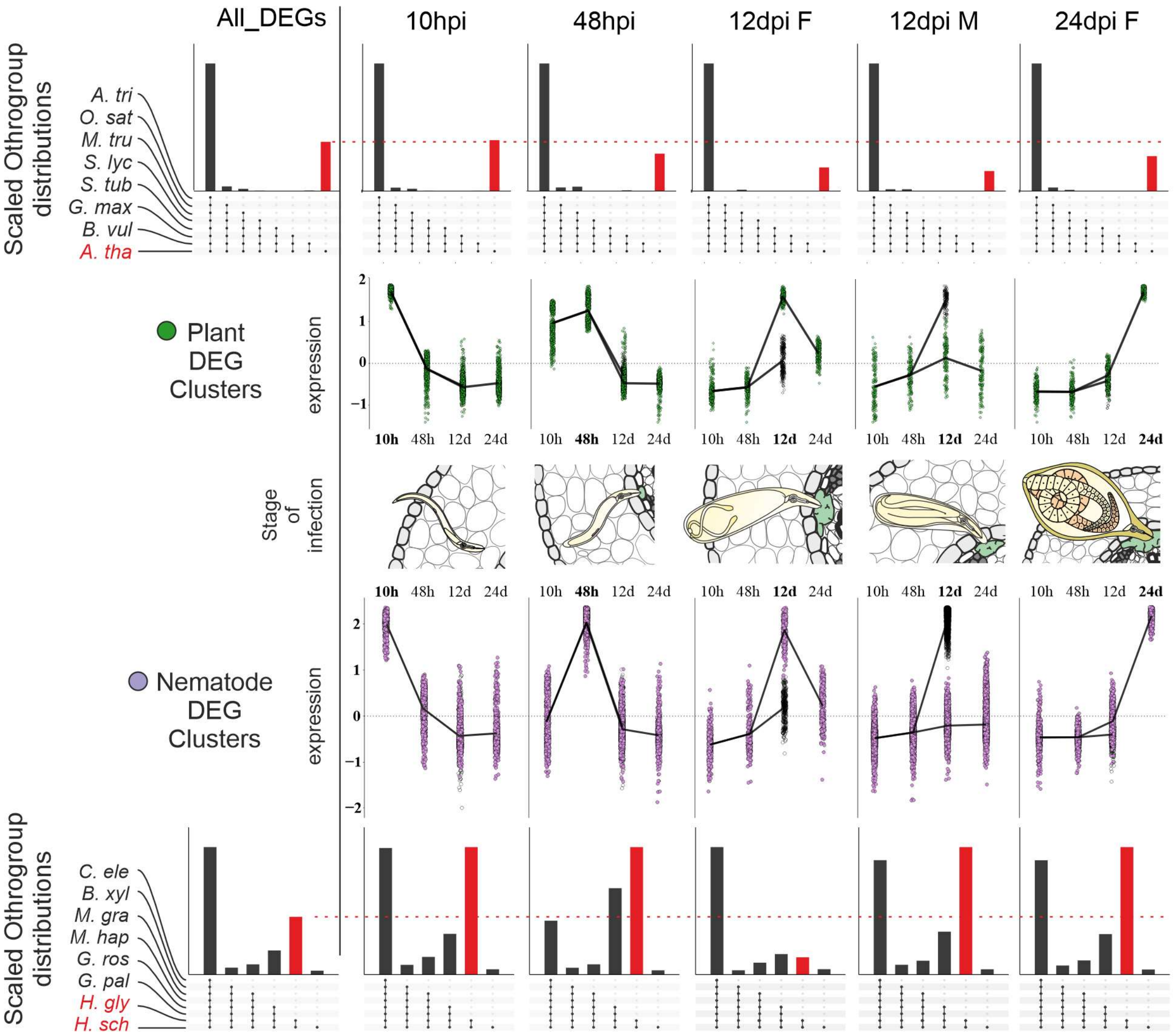

Supplemental Figure 5. Contrasting evolutionary histories of host and parasite genes deployed at specific times of infection. Differential expression super clusters that describe discrete stages of infection (centre) for either the host (green) or the parasite (purple). Upset graphs of orthologous gene clusters between eight nematode (bottom - *Caenorhabditis elegans, Bursaphelenchus xylophilus*, *Meloidogyne graminicola, M. hapla*, *Globodera pallida, G. rostochiensis*, *Heterodera glycines,* and *H. schachtii*) and eight plant species (top - *Amborella trichopoda, Oryza sativa, Medicago truncatula*, *Solanum lycopersicum, S. tuberosum*, *Glycine max*, *Beta vulgaris* and *A. thaliana*). Red highlights a subset of orthogroups for host and parasite.

Phylogeny of nematode PANC closest relatives

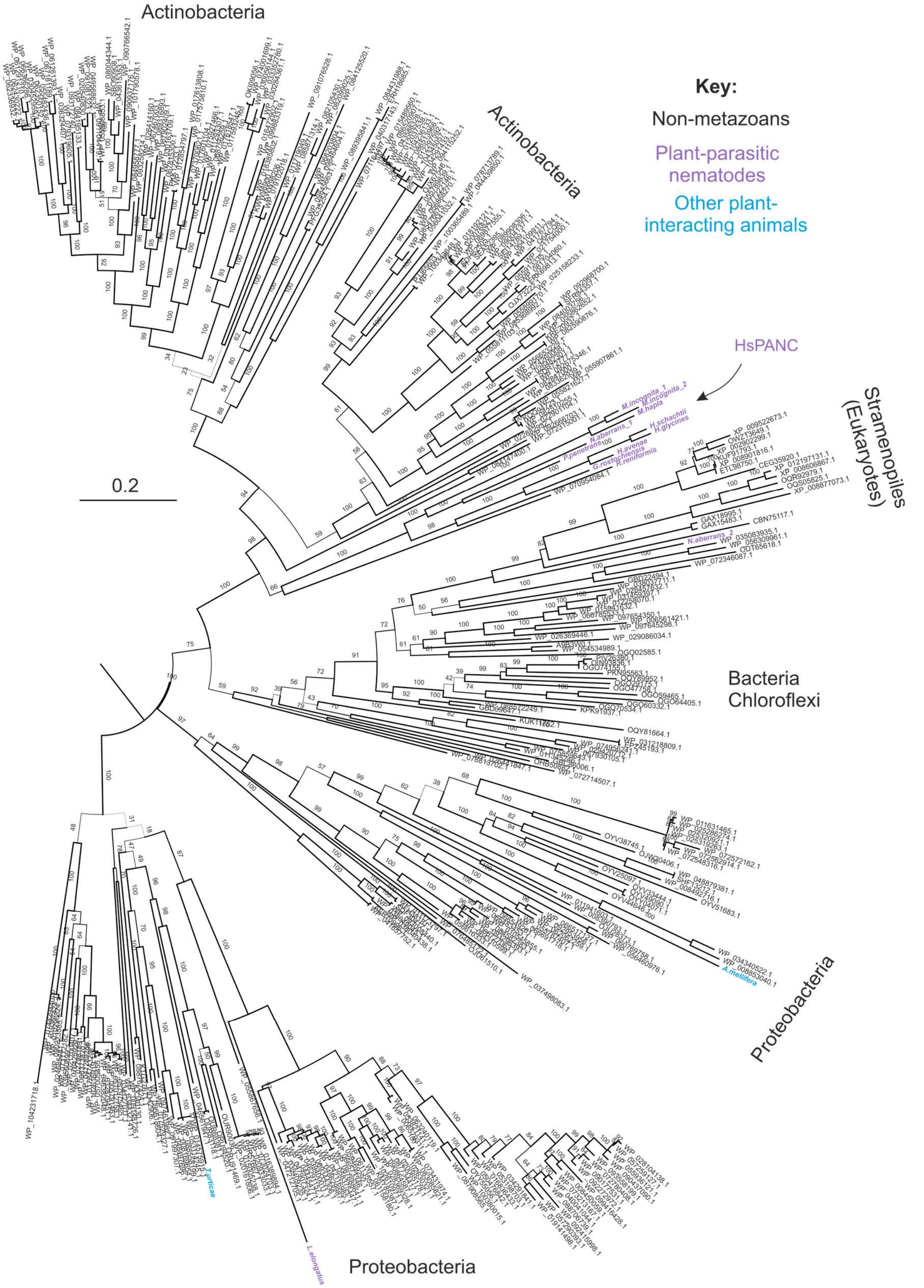

Supplemental Figure 6. A phylogeny of PANC-like sequences from plant-interacting organisms and non-Metazoans. The mid-point re-routed phylogenetic tree is inferred from the protein alignment of the top 50 most similar sequences identified in the NCBI non-redundant database to each of 14 animal putative PANC proteins. Most PANC homologues from plant-parasitic nematodes (purple), including Hs-PANC, group in a single monophyletic sub-clade with sequences from actinobacteria. *PANC* from other plant-interacting organisms are shown in light blue. Node labels indicate bootstrap support values**.**

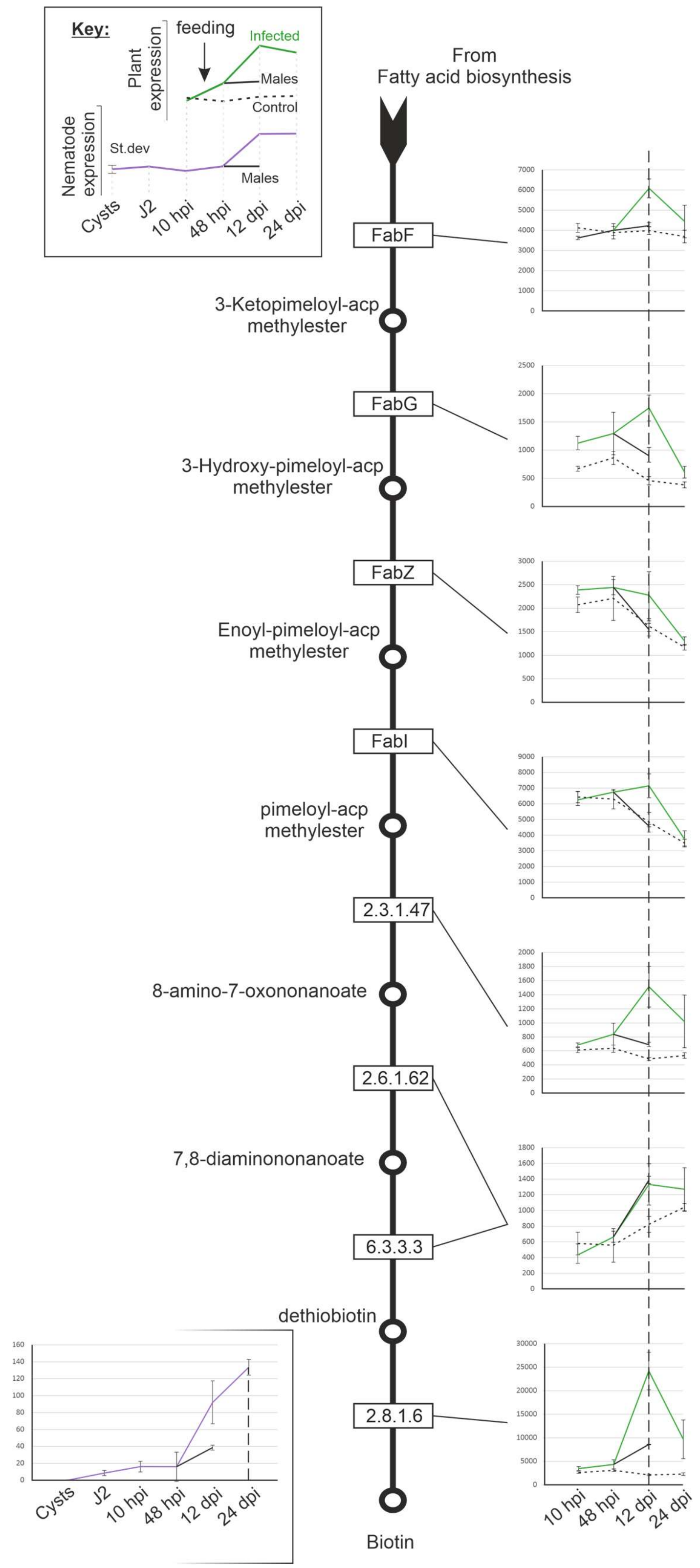

Supplemental Figure 7. The biotin biosynthesis pathway. Products/substrates are indicated with circles, enzymatic reactions (and corresponding EC codes) with squares. For each species, the expression profile of a gene annotated with this corresponding EC code is shown (in some cases there are multiple genes with the same EC code, Supplementary Table 6 and 7). Green for host, purple for parasite (if present). Error bars indicate standard deviation of the mean (center point). N =3 biologically independent samples.

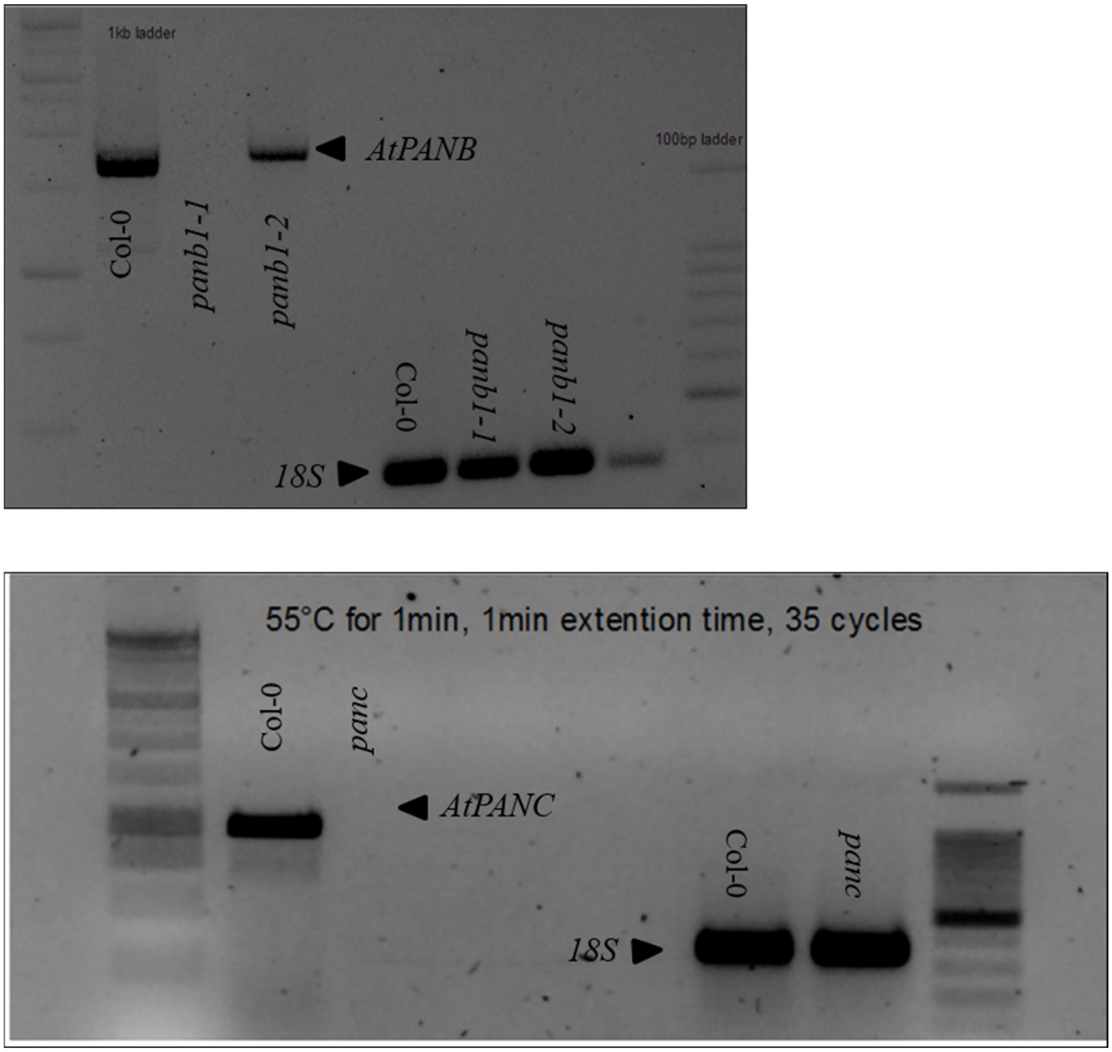

Supplemental Figure 8. RT-PCR for presence or absence of *AtPANB* and *AtPANC* expression in wild-type or loss-of functions mutant. RNA from Col-0, *atpanb1-1*, or *atpanc1* was extracted to synthesize single stranded cDNA. The presence or absence of expression is shown using primers given in Table S11. *18S* was used as a positive control.

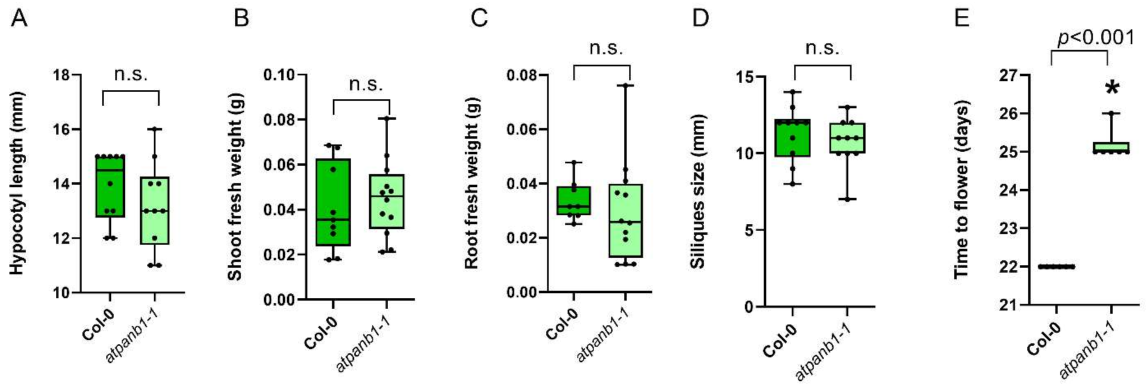

Supplemental Figure 9. Phenotypic analysis of the Col-0 and *atpanb1-1* mutant. **A**) Hypocotyl length of 5-days old plants (Col-0, $n = 10$; *atpanb1-1*, n=10). **B**) Shoot fresh weight of 12-days-old plants grown on Knop medium (Col-0, $n = 8$; *atpanb1-1*, n=11). **C**) Root fresh weight of 12-days-old plants grown on Knop medium (Col-0, $n = 8$; *atpanb1-1*, n=12). **D**) Silique length (Col-0, $n = 10$; *atpanb1-1*, n=10). (A-D) Data from three independent experiments is combined. E) Number of days to flower (Col-0, $n = 6$; *atpanb1-1*, n=6). Experiments were performed three times independently with the similar outcome. Data from one experiment is shown. D-E) Plants were grown on Knop medium for 6 days before they were transferred to soil in the greenhouse. A-E) Data were analysed using Student's *t*-test (two sided; α=0.05). No significant difference (n.s.) was detected. For box plots, centre line is median; box limits are upper and lower quartiles; whiskers are minimum and maximum value; points are all individual values superimposed on graphs. Source data are provided as a source data file.

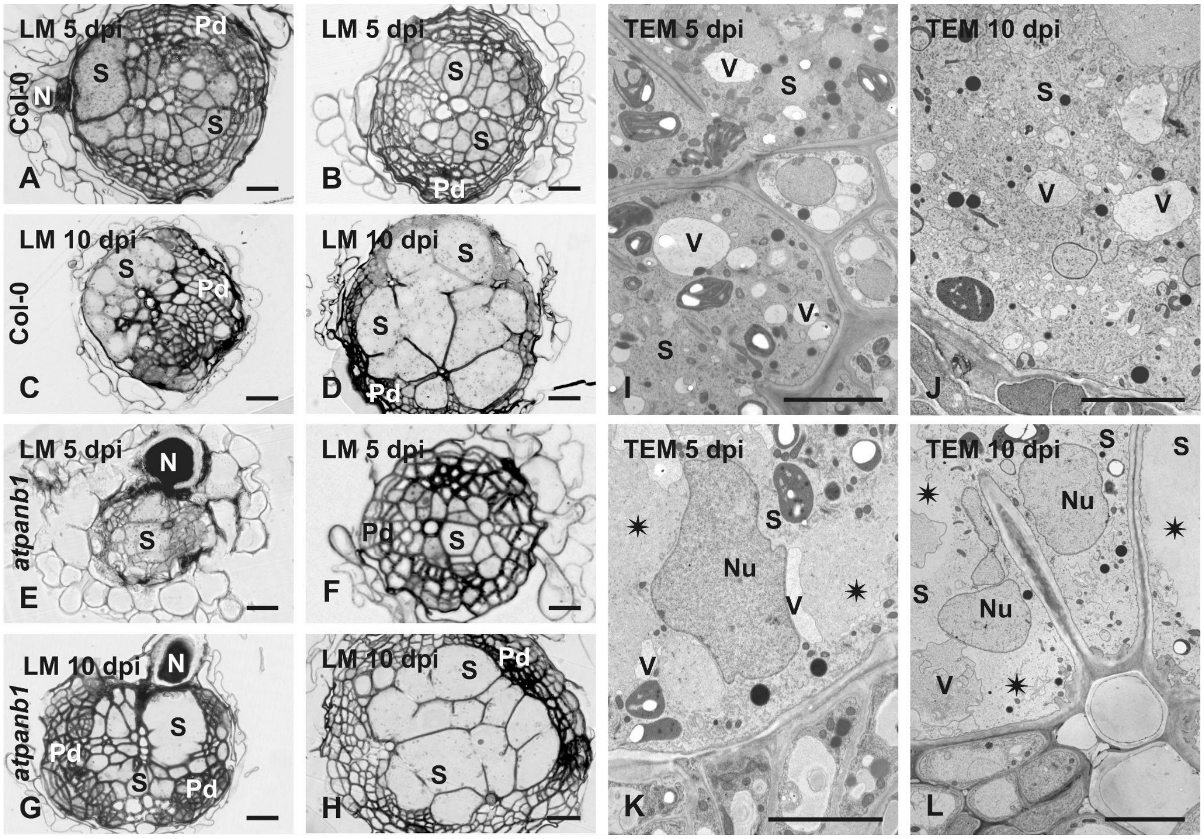

Supplemental Figure 10. Development of syncytia induced by *H. schachtii* in Col-0 and *atpanb1-1* roots. Light (**A**-**H**) and transmission electron microscopy (**I**-**L**) images of sections taken from syncytia induced in roots of Col-0 (**A**-**D**, **I**, **J**) and *atpanb1-1* plants (**E**-**H**, **K**, **L**) grown on Knop medium without vitamin B5. Sections were taken from samples collected 5 (**A**, **B**, **E**, **F**, **I**, **K**) and 10 dpi (**C**, **D**, **G**, **H**, **J**, **L**). Light microscopy images (**A**-**H**) were made from sections taken in close vicinity of juvenile heads (**A**, **C**, **E**, **G**) or in some distance from the heads in the widest part of the syncytium (**B**, **D**, **F**, **H**). Transmission electron microscopy images (**I**-**L**) were obtained from sections taken at the widest part of syncytium. Asterisks indicate organelles-free regions of syncytial cytoplasm in *atpanb1* plants (**K**, **L**). Abbreviations: N, nematode; Nu, nucleus; Pd, periderm; S, syncytium; V, vacuole. Scale bars: 20 μm (**A**-**H**) and 5 μm (**I**-**L**). Sectioning experiments were repeated twice with similar results. Each experiment consisted of several specimens that were sectioned (at least 5 per genotype). The representative images shown are from both experiments.

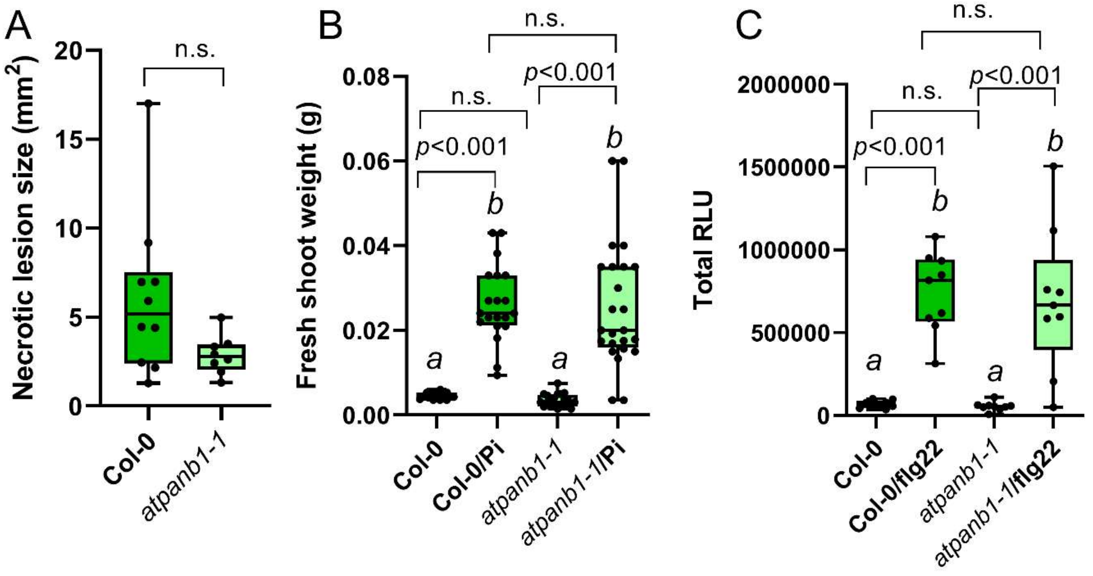

Supplemental Figure 11. Loss-of-function *atpanb1-1* is not impaired in plant immune responses. A) Infection assay with *Botrytis cinerea* under greenhouse conditions (Col-0, $n = 10$; *atpanb1-1*, n=8). Data were analysed using Student's *t*-test (two-sided; α=0.05). No significant difference (n.s.) was detected. Experiments were performed three times independently with the similar outcome. Data from one experiment is shown. **B**) Infection assay with *S. indica* for evaluation of its growth promoting effect. Experiments were performed three times independently and data from all three experiment is combined (Col-0, $n = 23$; Col-0/Pi, n=20 *atpanb1-1*, n=28; *atpanb1-1*/Pi, n=24). **C**) ROS burst in leaf disks treated with water or flg22 (n=9). ROS burst was measured by using L-012 based assay from 0 to 120 min. B and C) Data were analysed using a one-way analysis of variance (ANOVA) followed by Tukey's HSD post-hoc test (α=0.05). Columns not sharing the same letters indicate significantly different means. A-C) For box plots, centre line is median; box limits are upper and lower quartiles; whiskers are minimum and maximum value; points are all individual values superimposed on graphs. Source data are provided as a source data file.

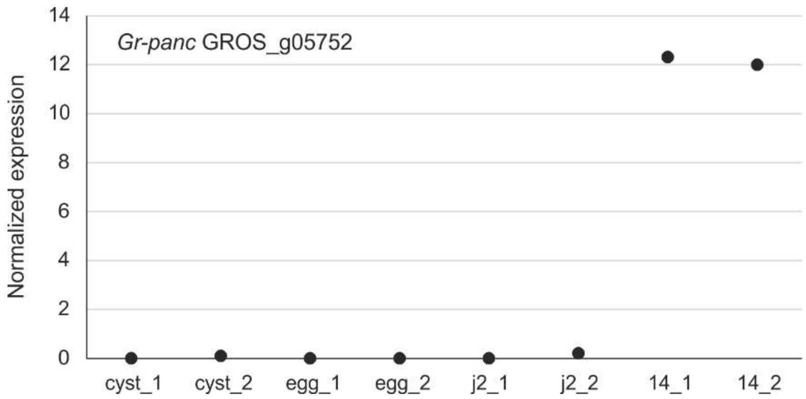

Supplemental Figure 12. *Gr-panc* Expression profile. Normalized expression values (from Eves-van den Akker, et al., 2016) for the corresponding homologue of *Hs-panc* (*Gr-panc*, GROS_g05752) for each replicate of four life stages is shown (cyst, egg, j2, and 14 days post infection).

```
Hs-panc1 - ATGTCGACCTCTTCAAAGTTGGCAGTGATCAACACCATTG - 40
Hs-panc2 - ATGTCGACCTCGTCAAAGTTGACAGTGATCAACACCATTG - 40

Hs-panc1 - CGAAAATGCGACAATTCGTGGGCGAACATCGTAAACAGAA - 80
Hs-panc2 - CGACAATGCGACAATTCGTGCGCGAACGTCGTAAACAGAA - 80

Hs-panc1 - CGCCAATCTTTGCGTGGCATTGGTGCCAACGATGGGTGCC - 120
Hs-panc2 - CGCCAATCTTTGCGTGGCATTGGTGCCGACAATGGGTGCT - 120

Hs-panc1 - CTTCACGAAGGCCATCTCCGACTTGCGCACCAGGCACGCC - 160
Hs-panc2 - CTTCACCAAGGCCATCTCCGACTGGCGCACCAGGCACGCT - 160

Hs-panc1 - TGTACAATAATGGTGAGGAACAAAATGGCACCACCGATCA - 200
Hs-panc2 - TGTACAAGGATGATGAGGAAAAAAGTGGCACAACTGATCA - 200

Hs-panc1 - GTGCGCCTCTCCTCTGCTGGTGATCGTCTCCATTTTTGTG - 240
Hs-panc2 - GTGCGCGTCTCTACCACTGGTGATTGTCTCCATTTTTGTG - 240

Hs-panc1 - AATCCGCTCCAATTTGGCGCAAACGAGGACTATGAGCGCT - 280
Hs-panc2 - AATCCGCTTCAATTTGGCCCAAACGAGGACTTTGAACGCT - 280

Hs-panc1 - ATCCGCGCACATTGGAAGCGGATTTGGCCAAATTGGAGGG - 320
Hs-panc2 - ATCCGCGCACATTAGAAGCGGATTTGGCCAAATTGGAGGG - 320

Hs-panc1 - CGTCGCGGATTGTGCATTTGTGCCAAATGCCGTGGAAATG - 360
Hs-panc2 - CGTCGCGGATTGTGCATTTGTGCCAAATGCCGTGGAAATG - 360

Hs-panc1 - TTCGGGGAGGAAAAGCAACCGCTGAAGGACCAGGACGAGG - 400
Hs-panc2 - TTCGGCGAGGAGAAGCAACCGCTGAAGCACCAGGACGAGG - 400
                                siRNA1

Hs-panc1 - ATGATGGCATCGGCATCAAATCCGGACGGATTGGCCGAAT - 440
Hs-panc2 - AGGATGGCATTGGCATCAAATCCGGACGGATTGGCCGAAT - 440
                                      Hs-panc1
                                      Hs-panc2

Hs-panc1 - TGTGGAGGGTGTCACACGACCGCTGTACCATGACGGAATG - 480
Hs-panc2 - TTTAGAGGGTGTCACACGACCACAGTACCATGACGGAATG - 480

Hs-panc1 - TTGTTGCATACGGTCAAACTTTACAACATTGCGCAACCAA - 520
Hs-panc2 - TTGCTGCATGTGGCCAAACTGTTCAACATTGTGCAACCAG - 520
                                          siRNA2

Hs-panc1 - ATTTGGTATTTTTCGGGAAAAAGGATGCGCAACAGCTGTT - 560
Hs-panc2 - ATTCGGCATTTTTCGGAAAAAAGGATGCTCAACAGCTGTT - 560

Hs-panc1 - CGCCATCCAACAAATGACCGCCGTCATGAATTTCCCGGTC - 600
Hs-panc2 - TGCCATCCAAGAAATGACCGTGGTCATGAATTTCCCGGTC - 600

Hs-panc1 - CATGTTGTGCCGGTAGACATTGAACGAGACCCGGACGGTT - 640
Hs-panc2 - CATGTTGTGCCGGTGGAAACTGAACGAGACCCGGACGGTT - 640

Hs-panc1 - TGGCACTGTCCAGCCGAAATCGGTTCTTAAGTGCCGAAGA - 680
Hs-panc2 - TGGCATTGTCAAGCCGAAATCAGTACTTAAGTGCCGAAGA - 680
             qPCR_F

Hs-panc1 - ACGGAGACGGGCATTGTGCCTGTACAATATGTTGGTGACG - 720
Hs-panc2 - ACGGCGACGCGCATTGTGCCTGTACAATATGTTGGTGACG - 720

Hs-panc1 - GCACGCAACGCCGCAACGAATGGTGCCAATTTGGCCTCAG - 760
Hs-panc2 - GCACGCGATGCCGCAACGAATGGTGCCAATTTGGCGTCAG - 760

Hs-panc1 - TGACGGCACAAGCGCAACAAATGTTAGCCCAGGCGGAAAA - 800
Hs-panc2 - TTGTGATAAAAGCTCACCAAATGCTGGCCCAAGCAGAAAA - 800

Hs-panc1 - GCAGGACGGCATAAAGACGGACTATTTTGCGGTGGTCGAC - 840
Hs-panc2 - GCAAGATGGCATAAAGACGGACTATTTTGCGGTGGTCGAC - 840
                                 qPCR_R

Hs-panc1 - CCGGACACATTCAGCCCATTGGAGGGCGACAAAATGGAGC - 880
Hs-panc2 - CCGGACACATTCAGCCCATTGGAGGACGACAAAATGGAGC - 880

Hs-panc1 - AGTTCAAAGGCAAAGCATTGCTTTTGGTCGCCGTTTTTGT - 920
Hs-panc2 - AGTTCAAAGGCAAAGCATTGCTTTTAGTCGCCGTTTTCAT - 920

Hs-panc1 - AGGCAGAATTCGGCTGATTGACAACATGGAAGTGCTAATT - 960
Hs-panc2 - GGGCAGAACTCGGCTGGTTGACAACATGGAAGTGCAAATC - 960

Hs-panc1 - AGTGGATAA                                - 969
Hs-panc2 - AGTGGATAA                                - 969
```

Supplemental Figure 13. CDS alignment of *Hs-panc1* and *Hs-panc2*. Highlighted are siRNA target sites (red) and qPCR primer sites (green) for *Hs-panc2* (Hsc_gene_23032.t1).

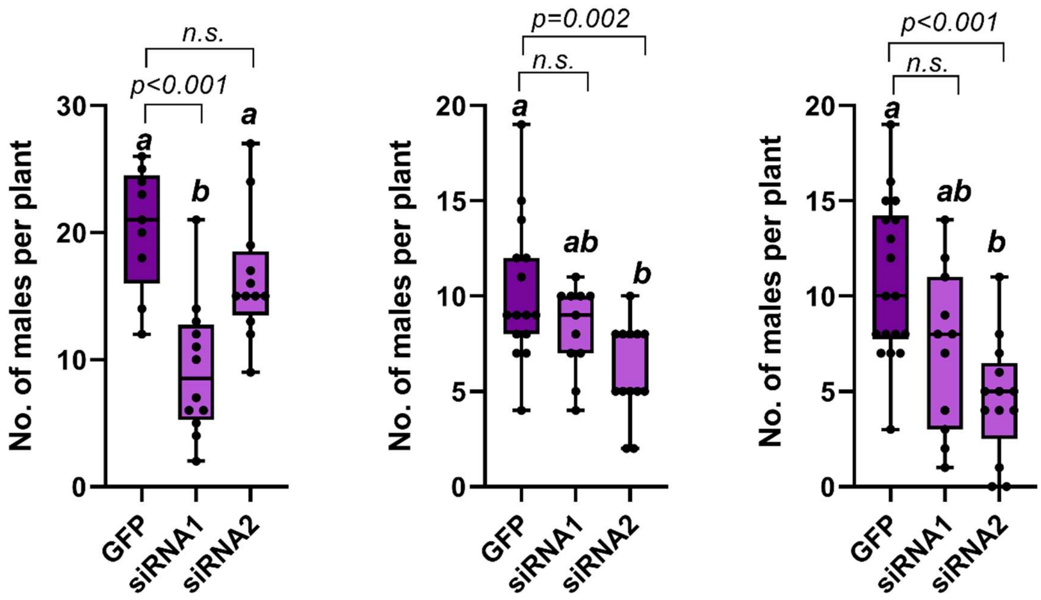

Supplemental Figure 14. Effect of silencing *Hspanc* expression on male number of cyst nematodes. Number of males present per root system at 14 dpi. Data from three independent experiments is shown (GFP, *n*=15, 9, 18; siRNA1, *n*=10, 11, 10; siRNA1, *n*=12, 11, 12). Data were analyzed using a one-way analysis of variance (ANOVA) followed by Tukey's HSD post-hoc test (α=0.05) and different letters indicate significantly different means. n.s. indicates no significant difference. Centre line is median; box limits are upper and lower quartiles; whiskers are minimum and maximum value; points are all individual values superimposed on graphs. Source data are provided as a source data file.

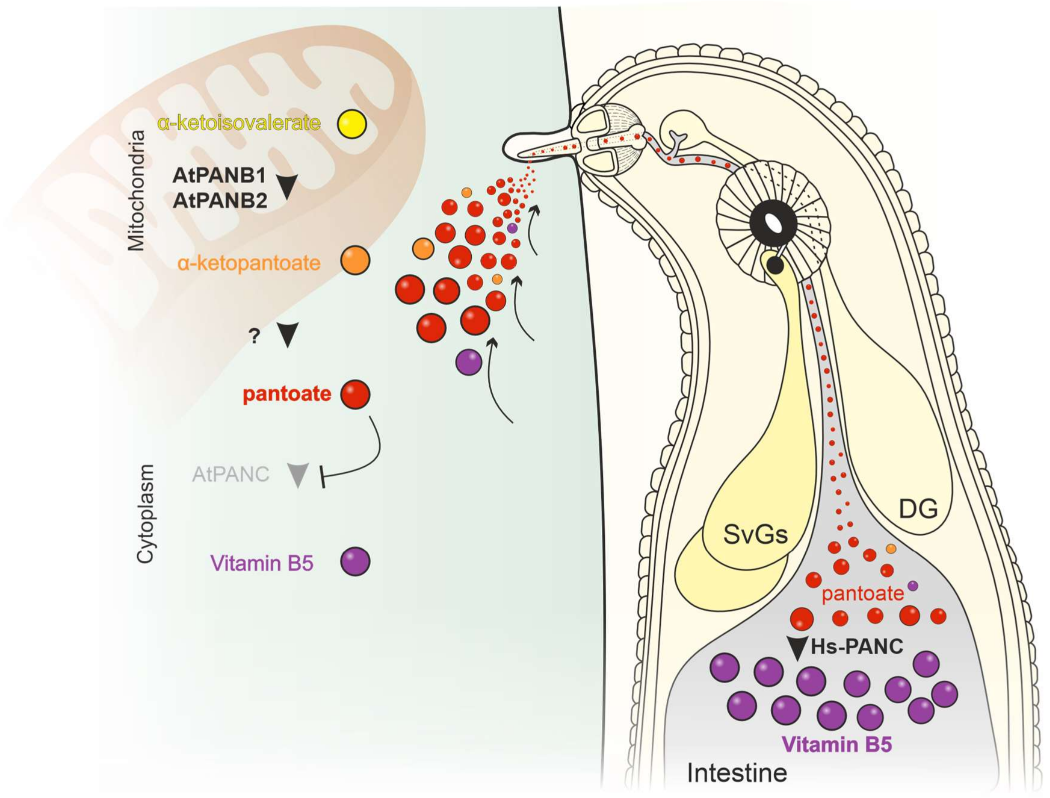

Supplemental Figure 15. A model for the trans-kingdom synthesis of vitamin B5. Left, plant cell. Right, parasitic nematode. The last two steps of the vitamin B5 biosynthesis pathway are indicated (bold names indicate upregulated during parasitism), including their subcellular localisation. Products/substrates are represented as globes. The working model is that the upregulation of all but the last step of vitamin B5 biosynthesis genes in the plant increases the concentration of pantoate. Various substrates/products of the pathway will be taken up by the nematode, albeit primarily pantoate. Pantoate is the substrate for Hs-PANC in the nematode: thus completing the trans-kingdom synthesis of vitamin B5.

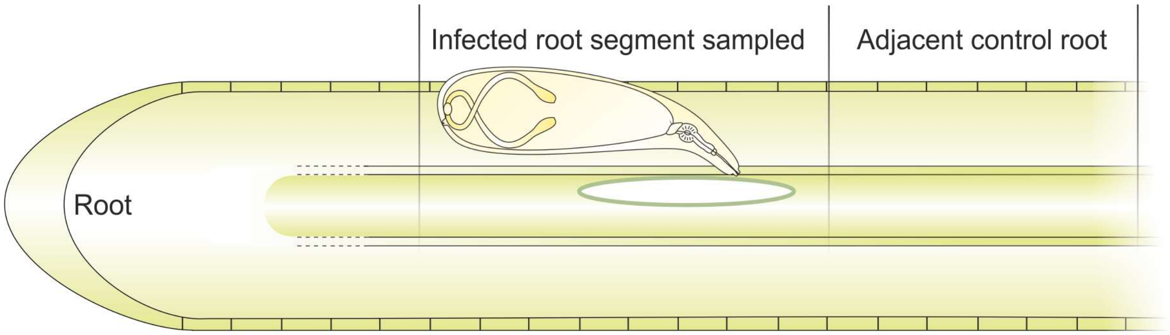

Supplemental Figure 16. Depiction of sample collection. Small root segments containing nematodes were marked under a stereo microscope. The infected area including nematodes was then hand-dissected (i.e. between the lines) and transferred to liquid nitrogen, avoiding confounding structures such as lateral roots and root tips. Adjacent (basipetal or acropetal) uninfected root segments, similarly avoiding confounding structures, were collected as control tissue. Several hundred such segments were collected for each biological replicate.

Supplementary Tables

Supplementary Table 1. Assembly statistics of the cyst nematodes.

	H. schachtii	*H. glycines*	*G. rostochiensis*	*G. pallida*	*G. ellingtonae*
**NUMBER OF SCAFFOLDS**	**395**	**9**	**173**	**6873**	**2246**
**GENOME SIZE (MB)**	**179**	**158**	**92**	**124**	**106**
**SCAFFOLD N50**	**1,273,070**	**17,908,190**	**1,700,000**	**120.481**	**327.189**
**NUMBER OF PREDICTED GENES**	**26.739**	**22.465**	**17.928**	**16.466**	**not released**
**REFERENCE**	**This paper**	**Masonbrink et al., 2021**[1]	**JJv, Steenbrugge et al., 2021.** [2]	**Cotton et al., 2014** [3]	**Phillips et al., 2017** [4]

Supplementary Table 2. BUSCO scores of plant-parasitic nematodes.

Species	BUSCO 5.1.2 nematoda_odb10								Accessions
	Assembly				Annotation				
	Sin	Dup	Frag	Mis	Sin	Dup	Frag	Mis	
Heterodera schachtii	55.3%	1.4%	2.1%	41.2%	53.5%	12.9%	2.2%	31.4%	
Heterodera glycines	52.8%	3.1%	2.2%	41.9%	51.5%	8.7%	1.5%	38.3%	GCA_004148225.2
Globodera pallida	43.9%	3.2%	2.3%	50.6%	41.9%	3.7%	2.8%	51.6%	PRJEB123
Globodera rostochiensis	58.6%	1.1%	1.8%	38.5%	64.3%	2.4%	2.1%	31.2%	PRJEB13504
Globodera ellingtonae	52.9%	1.6%	2.3%	43.2%	56.5%	8.9%	2.0%	32.6%	GCA_001723225.1
Meloidogyne hapla	58.3%	1.2%	2.1%	38.4%	51.7%	1.5%	2.6%	44.2%	PRJNA29083
Meloidogyne incognita	24.0%	39.8%	1.1%	35.1%	13.9%	54.6%	1.7%	29.8%	PRJEB8714
Meloidogyne arenaria	23.2%	38.3%	1.3%	37.2%	12.0%	56.1%	1.5%	30.4%	PRJNA438575
Meloidogyne enterolobii	34.2%	22.7%	2.7%	40.4%	28.6%	21.1%	3.6%	46.7%	PRJNA340324
Meloidogyne floridensis	50.2%	3.2%	2.8%	43.8%	38.9%	2.5%	2.7%	55.9%	PRJNA340324
Meloidogyne graminicola	51.1%	0.5%	3.5%	44.9%	53.7%	0.8%	2.8%	42.7%	PRJNA411966
Ditylenchus destructor	69.0%	2.0%	1.5%	27.5%	64.9%	2.1%	2.4%	30.6%	PRJNA312427
Ditylenchus dipsaci	56.9%	3.3%	2.0%	37.8%	46.7%	7.9%	4.3%	41.1%	PRJNA498219
Bursaphelenchus xylophilus	67.7%	1.4%	2.3%	28.6%	71.7%	1.8%	1.7%	24.8%	PRJEA64437

Supplementary Table 3. Possible marker genes most different at each life stage.

Gene id	Most highly DE at stage	Annotation
**AT3G44860**	10hpi	farnesoic acid carboxyl-O-methyltransferase
**AT5G56640**	48hpi	myo-inositol oxygenase 5
**AT5G50790**	12dpi fem	Nodulin MtN3 family protein
**AT5G50790**	12dpi male	Nodulin MtN3 family protein
**AT5G43360**	24dpi	phosphate transporter 1;3

Supplementary Table 4. Number and percentage of genes in differential expression clusters of the host.

Category	Number of genes	Percentage of genes	Percentage of clustered genes
**NA**	24482	74.6	NA
**10HPI_48HPI_12DPI MALE**	1306	4.0	16.6
**12DPI FEM._24DPI**	1230	3.7	15.6
**10HPI_48HPI**	1144	3.5	14.5
**10HPI**	790	2.4	10.0
**NOT CLUSTERED BUT DE**	485	1.5	NA
**12DPI FEM._12DPI MALE_24DPI**	351	1.1	4.5
**INCREASING**	329	1.0	4.2
**48HPI**	305	0.9	3.9
**12DPI_FEM**	273	0.8	3.5
**48HPI_12DPI MALE**	227	0.7	2.9
**24DPI**	205	0.6	2.6
**10HPI_12DPI FEM._24DPI**	202	0.6	2.6
**10HPI_48HPI_24DPI**	194	0.6	2.5
**12DPI FEM._12DPI MALE**	185	0.6	2.4
**10HPI_24DPI**	181	0.6	2.3
**DOWN_AT_12DPI FEM.**	162	0.5	2.1
**12DPI MALE_12DPI FEM._24DPI**	133	0.4	1.7
**12DPI_MALE**	106	0.3	1.3
**48HPI_12DPI FEM.**	88	0.3	1.1
**10HPI_12DPI MALE**	76	0.2	1.0
**DECREASING**	75	0.2	1.0
**48HPI_12DPI FEM._12DPI MALE**	72	0.2	0.9
**48HPI_24DPI**	47	0.1	0.6
**48HPI_12DPI MALE_24DPI**	47	0.1	0.6
**48HPI_12DPI FEM._24DPI**	33	0.1	0.4
**12DPI MALE_24DPI**	25	0.1	0.3
**10HPI_12DPI_FEM.**	23	0.1	0.3
**DOWN_AT_12DPI MALE**	22	0.1	0.3
**DOWN_AT_48HPI**	18	0.1	0.2
**DOWN_AT_24DPI**	17	0.1	0.2
**SUM**	32833	100	100

Supplementary Table 5. Number and percentage of genes in differential expression clusters of the parasite.

Category	Number of genes	Percentage of genes	Percentage of clustered genes
**NA**	8164	30.5	NA
**NOT CLUSTERED BUT DE**	3577	13.4	NA
**12DPI MALE**	2881	10.8	19.2
**CYST**	1328	5.0	8.9
**10HPI_48HPI**	1176	4.4	7.8
**CYST_J2**	1119	4.2	7.5
**INCREASING**	782	2.9	5.2
**48HPI**	779	2.9	5.2
**10HPI**	777	2.9	5.2
**24DPI**	683	2.6	4.6
**J2_10HPI**	679	2.5	4.5
**J2**	589	2.2	3.9
**12DPI FEM._12DPI MALE**	581	2.2	3.9
**12DPI FEMALE_24DPI**	566	2.1	3.8
**J2_10HPI_48HPI**	343	1.3	2.3
**10HPI_48HPI_12DPI FEM.**	323	1.2	2.2
**12DPI FEM._12DPI MALE_24DPI**	314	1.2	2.1
**DECREASING**	293	1.1	2.0
**12DPI FEM.**	287	1.1	1.9
**10HPI_12DPI MALE**	284	1.1	1.9
**J2_48HPI_12DPI MALE**	260	1.0	1.7
**48HPI_12DPI FEM._24DPI**	190	0.7	1.3
**10HPI_48HPI_12DPI FEM._24DPI**	190	0.7	1.3
**J2_12DPI_MALE**	157	0.6	1.0
**J2_12DPI FEM._24DPI**	108	0.4	0.7
**48HPI_12DPI FEM.12DPIMALE_24DPI**	100	0.4	0.7
**CYST_J2_10HPI_48HPI**	62	0.2	0.4
**48HPI_12DPI FEM.**	60	0.2	0.4
**10H_48H_12D FEM._12D MALE_24DPI**	51	0.2	0.3
**J2_10HPI_12DPI MALE**	36	0.1	0.2
**SUM**	26739	100	100

Supplementary Table 6. Expression of genes involved in biotin biosynthesis in the parasite.

EC CODE/NAME	KEGG	GENE ID	CYST	J2	10_HPI	48_HPI	12D_F	12D_M	24D_F
**2.8.1.6**	K01012	Hsc_gene_24092.t1	48	56	144	164	429	179	372
**2.8.1.6**	K01012	Hsc_gene_1769.t1	0	9	16	16	92	39	134

Supplementary Table 7. Expression of genes involved in biotin biosynthesis in the host.

			INFECTED					UNINFECTED			
**EC CODE/NAME**	KEGG	Gene ID	10_hpi	48_hpi	12d_f	12d_m	24	10	48	12days	24
**FAB G**	K00059	AT4G13180.1	15300	10618	6218	3831	5274	15355	13437	5310	3296
**FAB G**	K00059	AT1G24360.1	9954	9054	9098	7434	6512	7849	8347	7188	6747
**FAB G**	K00059	AT3G04000.1	171	177	531	917	1316	23	43	283	1086
**FAB G**	K00059	AT3G29250.2	44542	22713	4647	8994	3369	11296	15002	9255	4455
**FAB G**	K00059	AT3G03980.1	1124	1295	1747	917	608	671	861	457	384
**FAB G**	K00059	AT5G18210.1	340	372	689	719	578	456	480	646	637
**FAB I**	K00208	AT2G05990.1	6341	6802	7147	4609	3794	6432	6301	4826	3492
**FAB I**	K00208	AT2G05990.2	6341	6802	7147	4609	3794	6432	6301	4826	3492
**2.3.1.47**	K00652	AT5G04620.2	685	839	1512	692	1019	607	631	482	532
**2.8.1.6**	K01012	AT2G43360.1	3456	4344	24178	8556	9668	2613	3081	2111	2281
**FAB Z**	K02372	AT2G22230.1	1782	1777	1005	969	755	1638	1625	1098	836
**FAB Z**	K02372	AT5G10160.1	2388	2449	2278	1545	1303	2077	2209	1618	1171
**FABF**	K09458	AT2G04540.1	579	693	1057	923	987	588	600	709	922
**FABF**	K09458	AT1G74960.1	3623	4036	6081	4279	4442	4125	3888	3986	3693
**FABF**	K09458	AT1G74960.2	3623	4036	6081	4279	4442	4125	3888	3986	3693
**FABF**	K09458	AT1G74960.3	3623	4036	6081	4279	4442	4125	3888	3986	3693
**FABF**	K09458	AT5G46290.3	5609	5490	7494	5761	5638	7060	5651	5832	6008
**FABF**	K09458	AT5G46290.1	5609	5490	7494	5761	5638	7060	5651	5832	6008
**2.6.1.62 AND 6.3.3.3**	K19562	AT5G57590.1	450	665	1332	1389	1271	577	554	822	1039

Supplementary Table 8. Primers used in this study.

PRIMER NAME	PRIMER SEQUENCE 5' -3'
**LBB1**	GCGTGGACCGCTTGCTGCAACT
**LBP1.3**	ATTTTGCCGATTTCGGAAC
**ATPANB1_GW_FP**	GGGGACAAGTTTGTACAAAAAAGCAGGCTGCATGGCGTCCTCACTCACTAG
**ATPANB1_GW_R**	GGGGACCACTTTGTACAAGAAAGCTGGGTCTCACTTTGAAGGCTCCATGTTC
**HS_PANC_F1**	ACGGTTTGGCATTGTCAAGC
**HS_PANC_R1**	ACCACCGCAAAATAGTCCGT
**ACTIN-F**	CGTGACCTCACTGACTACCT
**ACTIN-R**	CGTAGCACAACTTCTCCTTG
**HSPANC_SIRNA1-F**	AACCGCUGAAGCACCAGGACGCCTGTCTC
**HSPANC_SIRNA1-R**	AACGTCCTGGTGCTTCGCGGCCTGTCTC
**HSPANC_SIRNA2-F**	AACATTGTGCAACCAGATTCGCCTGTCTC
**HSPANC_SIRNA2-R**	AACGAATCTGGTTGCACAATGCCTGTCTC
**ATPANC_F**	GCATGGAACTTGTGTTCTTG
**ATPANC_R**	ATTGCATTTGCCTACGCATAC

Supplementary Discussion

In our experience, nematoda datasets for BUSCO do not represent plant parasitic nematodes (PPN) well (which is why CEGMA is still used). Indeed, the latest nematoda BUSCO dataset (odb10) contains only 7 nematode species, three of which are Caenorhabditis species and no PPN or even tylencomporpha (a taxonomic group encompassing most PPN) are included. For that reason, most PPN genomes to date have been interrogated with BUSCO using eukaryota models in addition to CEGMA. In order to make our BUSCO analyses comparable to analyses performed on other PPN genomes, we used the same eukaryota dataset and version as well as parameters. Unfortunately, BUSCO scores are not a good indication of completeness of the assembly for many obligate biotrophs, like several PPN, because this type of organism tends to lose gene considered “indispensable”, as they are complemented by the host (compare biotrophic and non-biotrophic PPN below). Supplementary Table 2 shows that BUSCO scores of the *H. schachtii* genome and annotation are comparable to that of other obligate biotrophic plant-parasitic nematodes.

Supplementary references

1. Masonbrink, R. *et al.* The genome of the soybean cyst nematode (Heterodera glycines) reveals complex patterns of duplications involved in the evolution of parasitism genes. *BMC Genomics* **20**, 119 (2019).
2. van Steenbrugge, J. J. M. *et al.* Comparative genomics of two inbred lines of the potato cyst nematode Globodera rostochiensis reveals disparate effector family-specific diversification patterns. *BMC Genomics* **22**, 611 (2021).
3. Cotton, J. A. *et al.* The genome and life-stage specific transcriptomes of Globodera pallida elucidate key aspects of plant parasitism by a cyst nematode. *Genome Biol.* **15**, R43 (2014).
4. Phillips, W. S. *et al.* The Draft Genome of Globodera ellingtonae. *J. Nematol.* **49**, 127 (2017).
