# Supplementary material for: The genome and lifestage-specific transcriptomes of a plant-parasitic nematode and its host reveal susceptibility genes involved in trans-kingdom synthesis of vitamin B5
Source: Nat Commun. 2022 Oct 19;13:6190. doi: 10.1038/s41467-022-33769-w (PMC9582021; doi:10.1038/s41467-022-33769-w)
Supplement: Supplementary file 2 — Reporting Summary [file 41467_2022_33769_MOESM2_ESM.pdf]

nature portfolio

Corresponding author(s): Florian Grundler and Sebastian Eves-van den Akker

Last updated by author(s): Sep 21, 2022

Reporting Summary

Nature Portfolio wishes to improve the reproducibility of the work that we publish. This form provides structure for consistency and transparency in reporting. For further information on Nature Portfolio policies, see our Editorial Policies and the Editorial Policy Checklist.

Statistics

For all statistical analyses, confirm that the following items are present in the figure legend, table legend, main text, or Methods section.

n/a	Confirmed	
☐	☒	The exact sample size (*n*) for each experimental group/condition, given as a discrete number and unit of measurement
☐	☒	A statement on whether measurements were taken from distinct samples or whether the same sample was measured repeatedly
☐	☒	The statistical test(s) used AND whether they are one- or two-sided<br>*Only common tests should be described solely by name; describe more complex techniques in the Methods section.*
☒	☐	A description of all covariates tested
☐	☒	A description of any assumptions or corrections, such as tests of normality and adjustment for multiple comparisons
☐	☒	A full description of the statistical parameters including central tendency (e.g. means) or other basic estimates (e.g. regression coefficient) AND variation (e.g. standard deviation) or associated estimates of uncertainty (e.g. confidence intervals)
☐	☒	For null hypothesis testing, the test statistic (e.g. *F*, *t*, *r*) with confidence intervals, effect sizes, degrees of freedom and *P* value noted<br>*Give P values as exact values whenever suitable.*
☒	☐	For Bayesian analysis, information on the choice of priors and Markov chain Monte Carlo settings
☒	☐	For hierarchical and complex designs, identification of the appropriate level for tests and full reporting of outcomes
☒	☐	Estimates of effect sizes (e.g. Cohen's *d*, Pearson's *r*), indicating how they were calculated

Our web collection on statistics for biologists contains articles on many of the points above.

Software and code

Policy information about availability of computer code

Data collection	No software was used to collect data in this study.
Data analysis	As listed in the methods, the following software were used for data analysis: Canu snapshot v1.7 +137 changes (r8829 73d5caa1b1087b65f7853ecbebc1bb1dcbd1bc14), wtdbg2 Version: 2.1 - 20181007, BUSCO version 1.1b, Augustus version 3.2.1 models, STAR 2.7.0e, CEGMA v2.5, BlobTools version 1.0, minialign version 0.5.2, purge haplotigs, SSPACE_longreads, gapFinisher, smrttools-release_6.0.0.47835, Pilon, minimap2 v2.22, Repeatmodeler version DEV, RepBaseRepeatMaskerEdition-20170127, TransposonPSI version 08222010, LTRharvest 666 version 1.5.9 from Genometools, Braker2, DIAMOND-BLASTp version v0.9.24.125, BLAST2GO version 5, MCSanX toolkit, hmmsearch version 3.2.1, signalP 4.0, Phobius version 1.01, Whatshap v1.0, Samtools, Alienness web server, AvP (https://github.com/GDKO/AvP), MAFFT, FastTree, IQ-TREE 2, MUSCLE, Trimal, FastQC v0.11.8, BBduk v38.34, HTseq v0.12.4, R v3.5.2, DESeq2 v1.22.2, MATLAB v9.6 R2019a, tidyverse package v1.2.1, ggplot2 v3.1.0, KEGG Automatic Annotation Server Ver. 2.1.<br><br>Code and scripts used in the manuscript are available in the following repositories: https://github.com/sebastianevda/H.schachtii_genome, https://github.com/peterthorpe5/Heterodera_schachtii_genome_assembly, and https://github.com/OlafKranse/ALT_caller_for_pilon.

For manuscripts utilizing custom algorithms or software that are central to the research but not yet described in published literature, software must be made available to editors and reviewers. We strongly encourage code deposition in a community repository (e.g. GitHub). See the Nature Portfolio guidelines for submitting code & software for further information.

Data

Policy information about availability of data

All manuscripts must include a data availability statement. This statement should provide the following information, where applicable:

- Accession codes, unique identifiers, or web links for publicly available datasets
- A description of any restrictions on data availability
- For clinical datasets or third party data, please ensure that the statement adheres to our policy

The Whole Genome Shotgun data generated in this study have been deposited in the DDBJ/ENA/GenBank database under accession code JAHGVF010000000 (https://www.ncbi.nlm.nih.gov/nuccore/JAHGVF000000000.1). The Genomic and RNAseq data generated in this study have been deposited in the DDBJ/ENA/GenBank database under accession code Bioproject: PRJNA722882 (https://www.ncbi.nlm.nih.gov/search/all/?term=PRJNA722882). The Host expression data generated in this study are available in the Arabidopsis ePlant browser (https://bar.utoronto.ca/eplant/). The processed gene expression data generated in this study are available in the Supplementary Data associated with this manuscript. The Gene calls, proteins, etc. are available on wormbaseparasite (https://parasite.wormbase.org/index.html). Source data are provided with this paper.

Field-specific reporting

Please select the one below that is the best fit for your research. If you are not sure, read the appropriate sections before making your selection.

[x] Life sciences   [ ] Behavioural & social sciences   [ ] Ecological, evolutionary & environmental sciences

For a reference copy of the document with all sections, see nature.com/documents/nr-reporting-summary-flat.pdf

Life sciences study design

All studies must disclose on these points even when the disclosure is negative.

Sample size	No sample size calculations were performed. Sample sizes were determined by: a) technical tractability - for the extremely challenging sample collection for cross-kingdom transcriptomics, three repetitions was the most that could be reasonably achieved and because statistical difference calculations require at least two repetitions and take into account sample size and variance; and b) previous experience working with plant-parasitic nematode assays - for infection assays of H. schachtii on A. thaliana the standard in the field is 15-25 biological replicates per infection assay (e.g. Chopra, D., Hasan, M. S., Matera, C., Chitambo, O., Mendy, B., Mahlitz, S. V., ... & Siddique, S. (2021). Plant parasitic cyst nematodes redirect host indole metabolism via NADPH oxidase-mediated ROS to promote infection. New Phytologist, 232(1), 318-331).
Data exclusions	No data were excluded from the analyses
Replication	All attempts at replication were successful. Number of attempts of infection assays varies from 3 to 7. In each case repetitions are included in the source data file.
Randomization	Samples of tens of thousands of individuals (e.g. a population of nematodes to be treated with dsRNA) were divided equally into experimental conditions. Randomized allocation is impossible for such samples.
Blinding	The investigators were not blind to group allocation during data collection or analyses.

Reporting for specific materials, systems and methods

We require information from authors about some types of materials, experimental systems and methods used in many studies. Here, indicate whether each material, system or method listed is relevant to your study. If you are not sure if a list item applies to your research, read the appropriate section before selecting a response.

Materials & experimental systems

n/a	Involved in the study	
[x]	[ ]	Antibodies
[x]	[ ]	Eukaryotic cell lines
[x]	[ ]	Palaeontology and archaeology
[ ]	[x]	Animals and other organisms
[x]	[ ]	Human research participants
[x]	[ ]	Clinical data
[x]	[ ]	Dual use research of concern

Methods

n/a	Involved in the study	
[x]	[ ]	ChIP-seq
[ ]	[x]	Flow cytometry
[x]	[ ]	MRI-based neuroimaging

Animals and other organisms

Policy information about studies involving animals; ARRIVE guidelines recommended for reporting animal research

Laboratory animals	Experiments in this study were only performed on the following animal. Genus and species: Heterodera schachtii; strain: "Bonn"; sex: juvenile, male, and female; age: all stages of life cycle.
Wild animals	The study did not involve wild animals.
Field-collected samples	No field collected samples were used in this study. Nematodes were reared on plants grown in Petri dishes in agar supplemented with modified Knop's nutrient medium at 23 °C under long day conditions with 16 h light and 8 h darkness. Second stage juveniles were harvested from nematode stock culture, which has been maintained on mustard roots under sterile conditions for 20 years.
Ethics oversight	No ethical approval was required because Heterodera schachtii are invertebrate nematode worms.

Note that full information on the approval of the study protocol must also be provided in the manuscript.

Flow Cytometry

Plots

Confirm that:

- [x] The axis labels state the marker and fluorochrome used (e.g. CD4-FITC).
- [x] The axis scales are clearly visible. Include numbers along axes only for bottom left plot of group (a 'group' is an analysis of identical markers).
- [x] All plots are contour plots with outliers or pseudocolor plots.
- [x] A numerical value for number of cells or percentage (with statistics) is provided.

Methodology

Sample preparation	Flow cytometry was used to provide a rough estimation of genome size. Approximately 50 µl of compacted live H. schachtii J2s (Bonn population) in a 1.5 ml eppendorf tube were resuspended in 10 µl of extraction buffer (PBS with 0.01% Triton X) and disrupted with a micro pestle. Lysed nematodes were resuspended in 500 µl of extraction buffer and the debris filtered through 100 µm and then 30 µm filters (CellTrics). Up to 250 µl of the filtrate was added to 1 ml of staining buffer (100 mM Tris (pH 583 7.4), 150 mM NaCl, 1 mM CaCl2, 0.5 mM MgCl2, 2 µg ml-1 propidium iodide, and 0.18 mg ml-1 RNAse A) and incubated on ice in the dark for 1 h. For A. thaliana, 1-2 leaves were removed and placed on a Petri dish, immersed in 250-500 µl of extraction buffer. The leaf tissue was finely cut with a razor blade. The remaining liquid and leaf debris was filtered, stained, and counted as for nematode samples
Instrument	Accuri C6 flow cytometer (BD 585 Biosciences-US)
Software	FlowJo.
Cell population abundance	The purity of the samples was deemed low, given the crude nature of nuclei extraction.
Gating strategy	From crude extracts, a gate that incorporates propidium iodide stained nuclei was empirically derived using the characteristic and easily recognisable endo-reduplication ladder pattern of A. thaliana leaves (Galbraith, David W., and Guiling Sun. "Flow cytometry and sorting in Arabidopsis." Arabidopsis Protocols. Humana, New York, NY, 2021. 255-294) covering genome sizes from approx. 135 to 1080 mb. The same gate was then applied to H. schachtii. A figure exemplifying the gating strategy is provided in the Supplementary Information.

- [x] Tick this box to confirm that a figure exemplifying the gating strategy is provided in the Supplementary Information.
