# Supplementary material for: The genome and lifestage-specific transcriptomes of a plant-parasitic nematode and its host reveal susceptibility genes involved in trans-kingdom synthesis of vitamin B5
Source: Nat Commun. 2022 Oct 19;13:6190. doi: 10.1038/s41467-022-33769-w (PMC9582021; doi:10.1038/s41467-022-33769-w)
Supplement: Supplementary file 3 — Description of Additional Supplementary Files [file 41467_2022_33769_MOESM3_ESM.pdf]

File Name: Supplementary data 1

Description: Phased variant call file for *H. schachtii*.

File Name: Supplementary data 2

Description: All 601 sites in *H. schachtii* genome assembly Cam_Hsc_genome1.2 flagged by Pilon as being likely sites of larger structural variation.

File Name: Supplementary data 3

Description: Number of reads per RNAseq library and mapping statistics in each kingdom.

File Name: Supplementary data 4

Description: RNAseq expression for each *H. schachtii* gene during the infection time course.

File Name: Supplementary data 5

Description: High confidence effector predictions for *H. schachtii*.

File Name: Supplementary data 6

Description: Putative HGT events predicted in the *H. schachtii* gene calls.

File Name: Supplementary data 7

Description: RNAseq expression for each *A. thaliana* gene during the infection time course.

File Name: Supplementary data 8

Description: For each life stage specific expression cluster, the enrichment on each scaffold in the *H. schachtii* genome.

File Name: Supplementary data 9

Description: KEGG annotations for *H. schachtii* and *A. thaliana*.

File Name: Supplementary data 10

Description: All 3106 sites at which the second-most-abundant nucleotide call (QP score) was a tie between two different nucleotides, hence excluded from haplotype phasing but possibly representing second and third alleles.

File Name: Supplementary data 11

Description: All 3709 nucleotide sites at which more than two variants were present, possibly representing third alleles that are present at lower allele frequencies.

File Name: Supplementary data 12

Description: The two single haplotypes for each block of bi-allelic variants were placed (by random choice) into one of two genome-wide files to generate the two additional *H. schachtii* genome resources Haplotype1 and Haplotype2. This is Haplotype1.

File Name: Supplementary data 13

Description: The two single haplotypes for each block of bi-allelic variants were placed (by random choice) into one of two genome-wide files to generate the two additional *H. schachtii* genome resources Haplotype1 and Haplotype2. This is Haplotype2.
